# *EPAS1* and *VEGFA* gene variants are related to the symptoms of acute mountain sickness in Chinese Han population: a cross-sectional study

**DOI:** 10.1186/s40779-020-00264-6

**Published:** 2020-07-27

**Authors:** Ji-Hang Zhang, Yang Shen, Chuan Liu, Jie Yang, Yuan-Qi Yang, Chen Zhang, Shi-Zhu Bian, Jie Yu, Xu-Bin Gao, Lai-Ping Zhang, Jing-Bin Ke, Fang-Zheng-Yuan Yuan, Wen-Xu Pan, Zhi-Nian Guo, Lan Huang

**Affiliations:** Institute of Cardiovascular Diseases, Department of Cardiology, Xinqiao Hospital, Army Medical University, Chongqing, 400037 China

**Keywords:** Acute mountain sickness, Hypoxia, Single nucleotide polymorphism, Endothelial PAS domain protein 1 (EPAS1), Vascular endothelial growth factor (VEGFA)

## Abstract

**Background:**

More people ascend to high altitude (HA) for various activities, and some individuals are susceptible to HA illness after rapidly ascending from plains. Acute mountain sickness (AMS) is a general complaint that affects activities of daily living at HA. Although genomic association analyses suggest that single nucleotide polymorphisms (SNPs) are involved in the genesis of AMS, no major gene variants associated with AMS-related symptoms have been identified.

**Methods:**

In this cross-sectional study, 604 young, healthy Chinese Han men were recruited in June and July of 2012 in Chengdu, and rapidly taken to above 3700 m by plane. Basic demographic parameters were collected at sea level, and heart rate, pulse oxygen saturation (SpO_2_), systolic and diastolic blood pressure and AMS-related symptoms were determined within 18–24 h after arriving in Lhasa. AMS patients were identified according to the latest Lake Louise scoring system (LLSS). Potential associations between variant genotypes and AMS/AMS-related symptoms were identified by logistic regression after adjusting for potential confounders (age, body mass index and smoking status).

**Results:**

In total, 320 subjects (53.0%) were diagnosed with AMS, with no cases of high-altitude pulmonary edema or high-altitude cerebral edema. SpO_2_ was significantly lower in the AMS group than that in the non-AMS group (*P* = 0.003). Four SNPs in hypoxia-inducible factor-related genes were found to be associated with AMS before multiple hypothesis testing correction. The rs6756667 (*EPAS1*) was associated with mild gastrointestinal symptoms (*P* = 0.013), while rs3025039 (*VEGFA*) was related to mild headache (*P* = 0.0007). The combination of rs6756667 GG and rs3025039 CT/TT further increased the risk of developing AMS (*OR* = 2.70, *P* < 0.001).

**Conclusions:**

Under the latest LLSS, we find that *EPAS1* and *VEGFA* gene variants are related to AMS susceptibility through different AMS-related symptoms in the Chinese Han population; this tool might be useful for screening susceptible populations and predicting clinical symptoms leading to AMS before an individual reaches HA.

**Trial registration:**

Chinese Clinical Trial Registration, ChiCTR-RCS-12002232. Registered 31 May 2012.

## Background

Every year, millions of people ascend to high altitude (HA) above 2500 m for travel, work or military activities. However, some individuals are susceptible to HA illness or diseases, including acute mountain sickness (AMS), high-altitude pulmonary edema (HAPE) and high-altitude cerebral edema (HACE) [1]. AMS, a hypoxic syndrome, has characteristic symptoms, including headache, anorexia, dizziness, nausea, vomiting, fatigue, and sleep disturbance [2]. The incidence of AMS ranges from 10 to 85%, depending on the altitude reached, the ascension rate and individual susceptibility [3]. Most symptoms are self-limiting, and affected individuals recover after days of rest and acclimatization. Under severe conditions, some patients may develop life-threatening HAPE or HACE [4]. AMS has become a general complaint that limits activities of daily living and work capabilities at HA. Therefore, it is important to understand the parameters of susceptibility to AMS.

To date, the pathophysiology of AMS remains largely unknown. Although the primary etiology is believed to be hypobaric hypoxia, populations in highland regions, such as the Ethiopian Highlands, the Andes and the Tibetan Plateau, have a much lower risk of suffering from any mountain illness [5]. Interestingly, even under the same acute hypoxic conditions, certain lowland individuals are more susceptible to AMS than others. It is generally assumed that genetic background plays a pivotal role in the differences in susceptibility between lowlanders and highlanders [6]. Many genome-wide studies have revealed genetic variations in native highlanders related to adaptation at HA [5, 7–10]. Some important genes in the hypoxia-inducible factor (HIF) pathway are closely related to hypoxia adaptation in Tibetan and Andean populations [9]. Thus, genes involved in the HIF signaling pathway may be the basis for adaptive changes in plateau populations.

HIFs are transcription factors that directly or indirectly regulate hundreds of genes involved in angiogenesis, cell growth, apoptosis, energy metabolism and vasomotor regulation [11, 12]. HIF1α and HIF2α (also known as endothelial PAS domain-containing protein 1, EPAS1) are basic helix-loop-helix PAS domain transcription factors that are expressed in all metazoan organisms and are composed of α and β subunits [13]. Under hypoxic conditions, HIF1α regulates the transcription of hundreds of genes in a cell type-specific manner [14]. Hypoxia-sensitive gene pathways play key regulatory roles in red blood cell production (erythropoiesis), development, energy metabolism, angiogenesis, iron metabolism, heart and lung regulation and tumorigenesis in mammals [12]. HIF-1α accumulation and the subsequent upregulation of vascular endothelial growth factor (VEGF) potentially contribute to basement membrane damage, and exaggerated edema might be involved in the pathophysiology of AMS [15]. VEGF is a major angiogenic factor that plays an important role in regulating endothelial cell proliferation and vascular permeability, which has been shown to be markedly upregulated under hypoxic conditions. Two oxygen sensors, prolyl hydroxylase domain protein 2 (PHD2), also known as Egl nine homolog 1 (EGLN1), and factor inhibiting HIF-1α (FIH-1), also known as hypoxia-inducible factor 1-α inhibitor (HIF-1AN), play pivotal roles in regulating the HIF-1α pathway.

However, the role of polymorphisms in HIF pathway genes in the response to acute hypoxia, which may lead to AMS, has not been clarified. In a preliminary genome-wide association study of the Nepalese population, four single nucleotide polymorphisms (SNPs) in the *FAM149A* gene were found to be associated with AMS, but these results were not verified in two validation cohorts [16]. Moreover, few studies have examined the genetic component of susceptibility to AMS in lowlanders, such as the Chinese Han population, following acute exposure to HA. Our group showed that SNPs in *EPAS1* and the 5′-untranslated region (UTR) of *EGLN1* may be associated with a high risk of AMS [17, 18]. Ding et al. [19] reported a possible relationship between *VEGFA* polymorphisms and AMS in a relatively small Chinese Han population. In another study, *VEGFA* SNPs in transcription factor binding sites were analyzed and compared between relatively small Han Chinese and Tibetan populations (< 100), but no sites were associated with HA sickness, although some sites were associated with arterial oxygen saturation (SaO_2_) [20]. Since most of the Chinese Han population resides in the lowland region, they may be an appropriate population for investigating the association between genetic factors and susceptibility or resistance to AMS. Elucidating these associations may help millions of lowlanders predict their response to travel to plateaus and provide novel genetic targets for potential clinical intervention. Furthermore, no previous studies have reported genetic associations with AMS-related symptoms.

The AMS diagnosis system was recently revised by the Lake Louise AMS Score Consensus Committee in 2018 [21]. Based on two independent studies [22, 23] and suggestions from the consensus committee, disrupted sleep was excluded as a necessary symptom for the diagnosis of AMS [21]. It is unknown whether this exclusion will change our previous genetic findings regarding AMS. Thus, based on our field study of a relatively large population in 2012, we reevaluated 30 SNPs in 7 genes (*EGLN1, EGLN3, EPAS1, HIF1A, HIF1AN, PPARA,* and *VEGFA*) closely related to the HIF pathway according to the new 2018 scoring instructions and analyzed symptoms associated with the onset of AMS. We aimed to clarify the relationship between the genetic background of the Han population and the pathogenesis of AMS as well as related symptoms.

## Methods

### Subjects

A total of 604 unrelated Chinese Han males aged 18–45 years were recruited in June and July of 2012 in Chengdu (500 m above sea level). All subjects were military officers and soldiers who travelled from Chengdu to Lhasa (3700 m above sea level) by plane in 2 h. Health examinations were performed at Xinqiao Hospital (Chongqing, China) before the trip. Participants were enrolled only if they met the inclusion criteria of residing on the plain for generations without plateau exposure in the past 6 months. Subjects who suffered from diseases with similar clinical features to AMS, cardiovascular or respiratory system diseases, neurological diseases, cerebral vascular diseases, gastrointestinal system diseases or cancer, were excluded. Ethical approval was obtained from the Ethics Committee of Xinqiao Hospital, Army Medical University (number: 2012014; approved May 9, 2012). Each participant provided written informed consent for the collection of basic information, physical parameters and blood samples for further analyses. This study was registrated at Chinese Clinical Trial Registration (ChiCTR-RCS-12002232; Registered 31 May 2012).

### Data collection and AMS evaluation

Structured case report questionnaires were designed for participants to record their demographic information, including age, height, weight, body mass index (BMI), and smoking status, which was collected 7 days before travel. Physiological parameters, including heart rate (HR), pulse oxygen saturation (SpO_2_) and blood pressure (BP), and AMS-related symptoms, including headache, dizziness/light-headedness, gastrointestinal (GI) symptoms and fatigue, were collected 18–24 h after arriving in Lhasa. Participants rested for at least 10 min before physiological parameters were collected. A mercury sphygmomanometer was used to record HR and BP in the upper arm at heart level, and a Finger-Pulse Oximeter 503 (Criticare Systems, Inc., Waukesha, WI, USA) was used to record SpO_2_. AMS-related symptoms were recorded using the latest consensus of the 2018 Lake Louise scoring system (LLSS) [21], and subjects with a LLSS score ≥ 3 points and a headache were assigned to the AMS group, while those with a score ≥ 3 but no headache or a score < 3 were assigned to the non-AMS group.

### Selection of SNPs

Previous genome-wide association studies suggested that mutations from multiple genetic regions were associated with HA adaption between highlanders and lowlanders. We performed a search on the National Center for Biotechnology Information (NCBI) SNP database (http://www.ncbi.nlm.nih.gov/projects/SNP/) for basic information and the minor allele frequency (MAF) of selected SNPs in Chinese general population. Linkage disequilibrium (LD) analyses were performed and linkage maps were drawn using Haploview. As shown in Additional file 1, the correlation intensity between SNPs was evaluated by the values of LD coefficient (D’), logarithm of odds (LOD) and *r*^2^. Based on LD analyses, tag SNPs which represented neighboring SNPs in the genomic region were retained. In *EGLN1*, we selected rs2066140, rs508618, rs1538667, rs1339891 and rs2275279 which located in introns, rs2153364, rs1361384 and rs1339894 at 5′-UTR, rs12757362 and at the upstream of the gene [8, 10, 12, 18]. In *EPAS1*, we selected rs13419896, rs4953354 and rs6756667 in introns and rs1868092 at the downstream of the gene [7, 12, 19]. In *VEGFA*, we selected rs3025039 and rs10434 at 3′-UTR and rs1413711 in introns [20]. In *PPARA*, we selected rs4253623, rs135538, rs4253681 and rs4253747 in introns, rs7292407 which located 90 kbp from the transcriptional initiation site of gene [7, 8]. In *EGLN3*, we selected rs1680710 and rs11156819 at 3′-UTR [24]. In *HIF1A*, we selected rs2301104, rs12434438, rs2301112, rs2301113 and rs11549467 in introns, while in *HIF1AN*, we selected rs2295778 in introns, rs10883512 at 3′-UTR [9, 18]. In total, 30 tag SNPs on 7 HIF-related genes were included in this study.

### DNA extraction and genotyping

For DNA extraction, we collected approximately 5 ml of venous blood from each participant into EDTA tubes for anti-coagulation purposes. Genomic DNA was extracted from whole blood using the PAXgene Blood DNA kit (Qiagen, Hilden, Germany) and was stored at − 20 °C. Sequenom® Assay Design software (version 3.1, Sequenom Inc., San Diego, CA, USA) was used to design the polymerase chain reaction (PCR) primers listed in Additional file 2. After PCR amplification and purification, the DNA sequence containing the target SNP was subjected to single-base extension in a system with ddNTPs using iPLEX Gold technology. Then, the products were analyzed by matrix-assisted laser desorption ionization-time of flight mass spectrometry (MALDI-TOF MS) (Sequenom Inc., San Diego, CA, USA), and polymorphisms were genotyped on the basis of different detection peaks. All genotyping was performed in a blinded fashion, and 10% of the DNA samples were subjected to repeat detection.

### Statistical analysis

The statistical analysis was performed with SPSS software (version 24.0; SPSS, Inc., Chicago, IL, USA) and SNPStats online software (http://bioinfo.iconcologia.net/SNPstats). Continuous data were tested for normality using the Kolmogorov-Smirnov test. Variables with a normal distribution are presented as the mean ± standard deviation, and comparisons between groups were evaluated with the two-sided Student’s *t*-test. Variables that did not show a normal distribution are presented as medians and quartiles, and comparisons between groups were evaluated with the Mann-Whitney *U* test. Categorical variables are expressed as cases and percentages. The chi-square test was used to compare categorical variables, Hardy-Weinberg equilibrium (HWE) and allelic frequencies of SNPs between groups. Binary logistic regression was used to analyze associations between genotypes and AMS/AMS-related symptoms, which were further adjusted for potential confounders, including age, BMI and smoking status. Linkage disequilibrium analyses were performed with Haploview 4.2 software. False discovery rate (FDR) created by Benjamini and Hochberg was used for multiple hypothesis testing correction [25]. A two-sided *P* value < 0.05 indicated a statistically significant difference.

## Results

### Subject characteristics

**Fig. 1:**
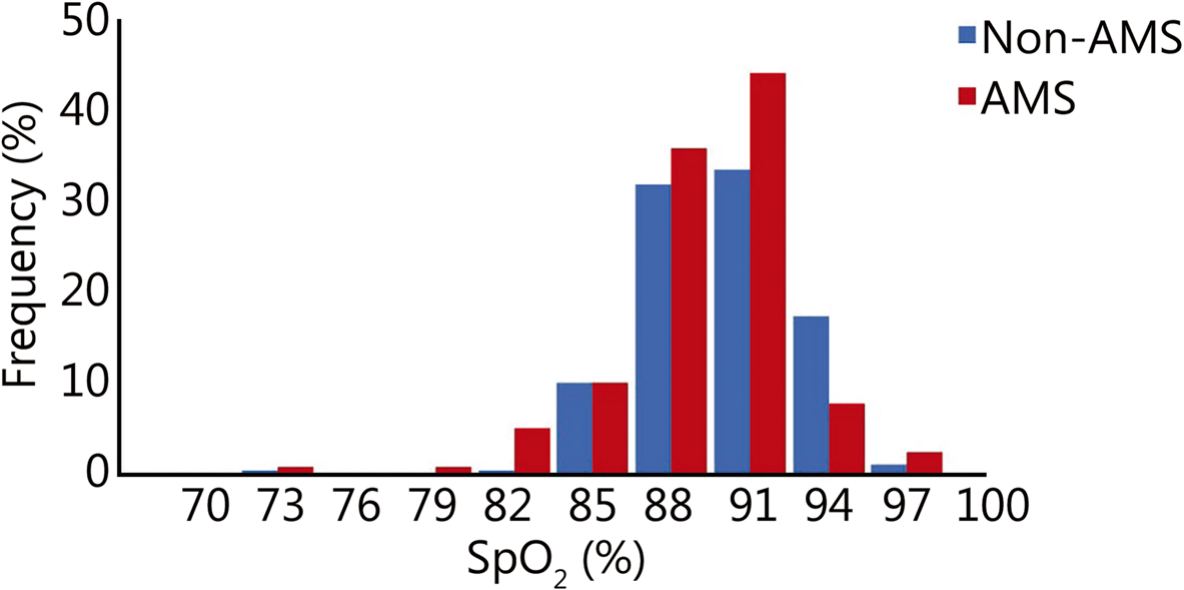
The frequency distribution histogram of SpO_2_ in non-AMS and AMS group. AMS. Acute mountain sickness; SpO_2_. Pulse oxygen saturation

Table 1 shows the basic characteristics of the subjects; 53.0% [*n* = 320; 23 (20, 25) years old] were diagnosed with AMS, and 47.0% [*n* = 284; 22 (20, 25) years old] were non-AMS. All participants were healthy Chinese Han soldiers who had lived in the plains for generations. No significant differences between groups were found in age, height, weight, body mass index (BMI), smoking status, HR, systolic BP (SBP) or diastolic BP (DBP). AMS patients had significantly lower SpO_2_ than non-AMS subjects [89% (86, 90) vs. 89% (87, 91), *P* = 0.003]. Although the mean values were close, the distribution frequency of SpO_2_ < 90% was higher in AMS than that in non-AMS group (66.2% vs. 56.0%, Fig. 1).

**Table 1:** Basic characteristics of the study groups (M(Q_1_, Q_3_))

Characteristics	Total (*n* = 604)	AMS group (*n* = 320)	Non-AMS group (*n* = 284)	*P*-value^a^
Basic demographic characteristics				
Age (year)	22 (20, 25)	23 (20, 25)	22 (20, 25)	0.210
Height (cm)	171 (168, 175)	171 (168, 174)	172 (168, 175)	0.343
Weight (kg)	64 (59, 68)	64 (59, 68)	63 (60, 68)	0.614
BMI (kg/m^2^)	21.6 (20.2, 22.8)	21.6 (20.2, 23.0)	21.5 (20.2, 22.6)	0.313
Smokers [*n* (%)]	322 (53.3)	161 (50.3)	161 (56.7)	0.117^b^
Physiological parameters				
HR (beat/min)	85 (78, 93)	85 (78, 94)	84 (77, 92)	0.072
SpO_2_ (%)	89 (87, 90)	89 (86, 90)	89 (87, 91)	0.003*
SBP (mmHg)	118 (110, 125)	119 (110, 126)	118 (110, 125)	0.500
DBP (mmHg)	79 (72, 85)	80 (72, 85)	78 (71, 85)	0.356

^a^ Mann-Whitney *U* test; ^b^*χ*^2^ test. * *P* < 0.05 indicted significant difference. *AMS* Acute mountain sickness; *BMI* Body mass index; *HR* Heart rate; *SpO*_*2*_ Pulse oxygen saturation; *SBP* Systolic blood pressure; *DBP* Diastolic blood pressure

### Associations between SNPs and AMS

Basic information of 30 SNPs in the *EGLN1*, *EPAS1*, *VEGFA*, *PPARA*, *HIF1A*, *HIF1AN* and *EGLN3* genes among subjects is presented in Additional file 3. Of these SNPs, rs1538667, rs1361384, rs1339894, rs12757362, rs1680710 and rs11549467 exhibited MAF less than 0.05, and the distribution of the rs1413711 G/A allele was not in HWE. Therefore, the above 7 SNPs were excluded, and further association analyses were conducted between the other 23 SNPs and AMS as well as AMS-related symptoms.

The exact mode of genetic inheritance was not clear, so we applied codominant, dominant and recessive models for genetic analyses. Ultimately, 4 SNPs (*EGLN1*-rs2153364, *EPAS1*-rs6756667, *VEGFA*-rs3025039 and *PPARA*-rs7292407) were found to be associated with the risk of developing AMS, even after adjusted by age, BMI and smoking status, as well as the results after FDR correction. The negative results of the other 19 SNPs were shown in Additional file 4.

The distributions of the *EGLN1*-rs2153364 A allele (AMS, 49.3%; non-AMS, 55.4%) and G allele (AMS, 50.7%; non-AMS, 44.6%) were significantly different between the AMS group and the non-AMS group (*P* = 0.044). Under the dominant model, the AG/GG genotype was associated with significantly increased AMS risk (*OR* = 1.56; 95% CI 1.07–2.28; *P* = 0.021) after adjusting for age, BMI and smoking status. However, the result was not significant after FDR correction (*Q* = 0.242). No significant results were found under the codominant or recessive model (Table 2).

**Table 2:** Associations analyses between *EGLN1*-rs2153364 and AMS

Model	Allele/Genotype	AMS group (*n* = 320)	Non-AMS group (*n* = 284)	*OR* (95% CI)	*P*-value	*OR* (95% CI)^a^	*P*-value^a^	*Q*-value
Allele	A	285 (49.3)	288 (55.4)	–	0.044*	–	–	–
	G	293 (50.7)	232 (44.6)	–		–		
Codominant	AA	69 (23.9)	84 (32.3)	1	0.085	1	0.068	0.391
	AG	147 (50.9)	120 (46.2)	1.49 (1.00–2.22)		1.53 (1.02–2.29)		
	GG	73 (25.2)	56 (21.5)	1.59 (0.99–2.54)		1.62 (1.01–2.61)		
Dominant	AA	69 (23.9)	84 (32.3)	1	0.028*	1	0.021*	0.242
	AG/GG	220 (76.1)	176 (67.7)	1.52 (1.05–2.21)		1.56 (1.07–2.28)		
Recessive	AA/AG	216 (74.7)	204 (78.5)	1	0.300	1	0.300	0.531
	GG	73 (25.3)	56 (21.5)	1.23 (0.83–1.83)		1.23 (0.83–1.84)		

^a^ adjusted for age, BMI and smoking status. * *P* < 0.05 indicted significant difference. “-” indicated “not available” for regression analysis or multiple hypothesis testing correction. *Q*-value was calculated using Benjamini and Hochberg method in multiple hypothesis testing including 23 SNPs for AMS association analysis. *SNP* Single nucleotide polymorphism; *AMS* Acute mountain sickness; *BMI* Body mass index; *OR* Odds ratio; *CI* Confidence interval

The distributions of the *EPAS1*-rs6756667 G allele (AMS, 91.1%; non-AMS, 85.0%) and A allele (AMS, 8.9%; non-AMS, 15.0%) were significantly different between groups (*P* = 0.001). Compared with individuals carrying the G allele, those carrying the A allele had a significantly decreased risk of AMS. The genotype frequencies of GG, GA and AA were 82.2% (*n* = 263), 17.8% (*n* = 57) and 0.0% (*n* = 0), respectively, in the AMS group and 72.2% (*n* = 205), 25.7% (*n* = 73) and 2.1% (*n* = 6), respectively, in the non-AMS group (*P* = 0.0005) and retained significance after FDR (*Q* = 0.012). Under the dominant model, the GA/AA genotype was associated with decreased AMS risk (*OR* = 0.56; 95% CI 0.38–0.82; *P* = 0.003) after adjusted for age, BMI and smoking status. FDR testing turned out to be insignificant (*Q* = 0.069) (Table 3).

**Table 3:** Associations analyses between *EPAS1*-rs6756667 and AMS

Model	Allele/Genotype	AMS group (*n* = 320)	Non-AMS group (*n* = 284)	*OR* (95% CI)	*P*-value	*OR* (95% CI)^a^	*P*-value^a^	*Q*-value
Allele	G	583 (91.1)	483 (85.0)	–	0.001*	–	–	–
	A	57 (8.9)	85 (15.0)	–		–		
Codominant	GG	263 (82.2)	205 (72.2)	1	< 0.001*	1	0.0005*	0.012*
	GA	57 (17.8)	73 (25.7)	0.61 (0.41–0.90)		0.60 (0.41–0.90)		
	AA	0 (0.0)	6 (2.1)	0.00 (0.00-NA)		0.00 (0.00-NA)		
Dominant	GG	263 (82.2)	205 (72.2)	1	0.003*	1	0.003*	0.069
	GA/AA	57 (17.8)	79 (27.8)	0.56 (0.38–0.83)		0.56 (0.38–0.82)		
Recessive	GA/GG	320 (100.0)	278 (97.9)	1	0.003*	1	0.003*	0.069
	AA	0 (0.0)	6 (2.1)	0.00 (0.00-NA)		0.00 (0.00-NA)		

^a^ adjusted for age, BMI and smoking status. * *P* < 0.05 indicted significant difference. “-” indicated “not available” for regression analysis or multiple hypothesis testing correction. *Q*-value was calculated using Benjamini and Hochberg method in multiple hypothesis testing including 23 SNPs for AMS association analysis. *SNP* Single nucleotide polymorphism; *AMS* Acute mountain sickness; *BMI* Body mass index; *OR* Odds ratio; *CI* Confidence interval

The frequencies of the *VEGFA*-rs3025039 C allele (AMS, 82.2%; non-AMS, 87.5%) and T allele (AMS, 17.8%; non-AMS, 12.5%) were significantly different (*P* = 0.011). The frequencies of the CC, CT and TT alleles were 67.3% (*n* = 214), 29.9% (*n* = 95) and 2.8% (*n* = 9), respectively, in the AMS group and 75.7% (*n* = 215), 23.6% (*n* = 67) and 0.7% (*n* = 2), respectively, in the non-AMS group. Under the dominant model, the CT/TT genotype was associated with increased AMS risk (*OR* = 1.51; 95% CI 1.05–2.16; *P* = 0.024) after adjusting for age, BMI and smoking status, but insignificant after FDR (*Q* = 0.184). No significant results were found under the recessive model (Table 4).

**Table 4:** Associations analyses between *VEGFA*-rs3025039 and AMS

Model	Allele/Genotype	AMS group (*n* = 320)	Non-AMS group (*n* = 284)	*OR* (95% CI)	*P*-value	*OR* (95% CI)^a^	*P*-value^a^	*Q*-value
Allele	C	523 (82.2)	497 (87.5)	–	0.011*	–	–	–
	T	113 (17.8)	71 (12.5)	–		–		
Codominant	CC	214 (67.3)	215 (75.7)	1	0.021*	1	0.022*	0.253
	CT	95 (29.9)	67 (23.6)	1.42 (0.99–2.05)		1.42 (0.98–2.05)		
	TT	9 (2.8)	2 (0.7)	4.52 (0.97–21.17)		4.56 (0.97–21.49)		
Dominant	CC	214 (67.3)	215 (75.7)	1	0.022*	1	0.024*	0.184
	CT/TT	104 (32.7)	69 (24.3)	1.51 (1.06–2.17)		1.51 (1.05–2.16)		
Recessive	CT/CC	309 (97.2)	282 (99.3)	1	0.042	1	0.042	0.483
	TT	9 (2.8)	2 (0.7)	4.11 (0.88–19.16)		4.15 (0.88–19.48)		

^a^ adjusted for age, BMI and smoking status. * *P* < 0.05 indicted significant difference. “-” indicated “not available” for regression analysis or multiple hypothesis testing correction. *Q*-value was calculated using Benjamini and Hochberg method in multiple hypothesis testing including 23 SNPs for AMS association analysis. *SNP* Single nucleotide polymorphism; *AMS* Acute mountain sickness; *BMI* Body mass index; *OR* Odds ratio; *CI* Confidence interval

As for *PPARA*-rs7292407, the allelic frequency was significantly different between the two groups (*P* = 0.014). Compared with individuals carrying the C allele, those carrying the A allele showed a significantly decreased risk of developing AMS. Under the dominant model, the AC/AA genotype was associated with decreased AMS risk (*OR* = 0.68; 95% CI 0.47–0.99; *P* = 0.046) after adjusting, but insignificant after FDR (*Q* = 0.265). And no significant results were obtained with the codominant or recessive model (Table 5).

**Table 5:** Associations analyses between *PPARA*-rs7292407 and AMS

Model	Allele/Genotype	AMS group (*n* = 320)	Non-AMS group (*n* = 284)	*OR* (95% CI)	*P*-value	*OR* (95% CI)^a^	*P*-value^a^	*Q*-value
Allele	C	524 (87.3)	445 (82.1)	–	0.014*	–	–	–
	A	76 (12.7)	97 (17.9)	–		–		
Codominant	CC	230 (76.7)	187 (69.0)	1	0.050	1	0.068	0.313
	AC	64 (21.3)	71 (26.2)	0.73 (0.50–1.08)		0.73 (0.50–1.09)		
	AA	6 (2.0)	13 (4.8)	0.38 (0.14–1.01)		0.40 (0.15–1.07)		
Dominant	CC	230 (76.7)	187 (69.0)	1	0.039*	1	0.046*	0.265
	AC/AA	70 (23.3)	84 (31.0)	0.68 (0.47–0.98)		0.68 (0.47–0.99)		
Recessive	CC/AC	294 (98.0)	258 (95.2)	1	0.061	1	0.083	0.318
	AA	6 (2.0)	13 (4.8)	0.41 (0.15–1.08)		0.43 (0.16–1.16)		

^a^ adjusted for age, BMI and smoking status. * *P* < 0.05 indicted significant difference. “-” indicated “not available” for regression analysis or multiple hypothesis testing correction. *Q*-value was calculated using Benjamini and Hochberg method in multiple hypothesis testing including 23 SNPs for AMS association analysis. *SNP* Single nucleotide polymorphism; *AMS* Acute mountain sickness; *BMI* Body mass index; *OR* Odds ratio; *CI* Confidence interval

### Associations between SNPs and AMS-related symptoms

Binary logistic regression analyses were conducted, and the dominant model was chosen to show correlations between SNPs and AMS-related symptoms (Table 6). Under the dominant model, subjects with the *EPAS1*-rs6756667 GA/AA genotype were less likely to suffer from AMS-related GI symptoms (*OR* = 0.53; 95% CI 0.32–0.89; *P* = 0.013), and the result was marginally significant after FDR correction (*Q* = 0.052). Meanwhile, *VEGFA*-rs3025039 was associated with AMS-related headaches. Under the dominant model, the rs3025039 CT/TT genotype showed a 2.08-fold increased risk of AMS-related headaches (*P* = 0.0007), and the results remained significant after FDR correction (*Q* = 0.028). No associations were found for *EGLN1*-rs2153364 and *PPARA*-rs7292407 with any AMS-related symptoms even before FDR testing. Based upon our findings with AMS-related symptoms, only *EPAS1*-rs6756667 and *VEGFA*-rs3025039 were applied for the following analysis. Further information, including the distribution of SNP alleles/genotypes and results under the recessive model, was presented in Additional files 5-8.

**Table 6:** Associations analyses between SNPs and AMS-related symptoms

SNP ID	Gene	Genotype	HD			DL			GI			FA		
			*OR* (95% CI) *OR* (95% CI)^a^	*P*-value *P*-value^a^	*Q*-value	*OR* (95% CI) *OR* (95% CI)^a^	*P*-value *P*-value^a^	*Q*-value	*OR* (95% CI) *OR* (95% CI)^a^	*P*-value*P*-value^a^	*Q*-value	*OR* (95% CI) *OR* (95% CI)^a^	*P*-value*P*-value^a^	*Q*-value
rs2153364	*EGLN1*	AA	1.26 (0.84–1.90)	0.270	0.250	1.39 (0.93–2.08)	0.110	0.098	1.29 (0.81–2.06)	0.270	0.540	1.26 (0.85–1.87)	0.250	0.220
		AG/GG	1.28 (0.84–1.93)	0.250		1.41 (0.94–2.11)	0.098		1.29 (0.81–2.07)	0.270		1.28 (0.86–1.91)	0.220	
rs6756667	*EPAS1*	GG	0.70 (0.46–1.05)	0.091	0.117	0.68 (0.45–1.03)	0.070	0.154	0.54 (0.32–0.90)	0.014*	0.052	0.69 (0.46–1.03)	0.070	0.264
		GA/AA	0.69 (0.46–1.05)	0.088		0.69 (0.46–1.04)	0.077		0.53 (0.32–0.89)	0.013*		0.68 (0.46–1.02)	0.066	
rs3025039	*VEGFA*	CC	2.08 (1.34–3.23)	< 0.001*	0.028*	1.40 (0.93–2.10)	0.100	0.107	0.99 (0.65–1.52)	0.960	0.950	1.42 (0.96–2.11)	0.073	0.178
		CT/TT	2.08 (1.34–3.23)	0.0007*		1.43 (0.95–2.16)	0.080		0.99 (0.64–1.51)	0.950		1.40 (0.95–2.08)	0.089	
rs7292407	*PPARA*	CC	0.68 (0.45–1.01)	0.058	0.160	0.71 (0.48–1.05)	0.091	0.264	0.80 (0.50–1.26)	0.330	0.493	0.72 (0.49–1.06)	0.094	0.160
		AC/AA	0.70 (0.46–1.04)	0.080		0.68 (0.46–1.02)	0.066		0.81 (0.51–1.29)	0.370		0.73 (0.49–1.08)	0.120	

^a^ adjusted for age, BMI and smoking status. * *P* < 0.05 indicted significant difference. *Q*-value was calculated using Benjamini and Hochberg method in multiple hypothesis testing including above 4 SNPs. *AMS* Acute mountain sickness; *HD* Headache; *DL* Dizziness and light-headedness; *GI* Gastrointestinal symptoms; *FA* Fatigue and/or weakness; *OR* Odds ratio; *CI* Confidence interval

### Associations between SNPs and the intensity of AMS/AMS-related symptoms

**Fig. 2:**
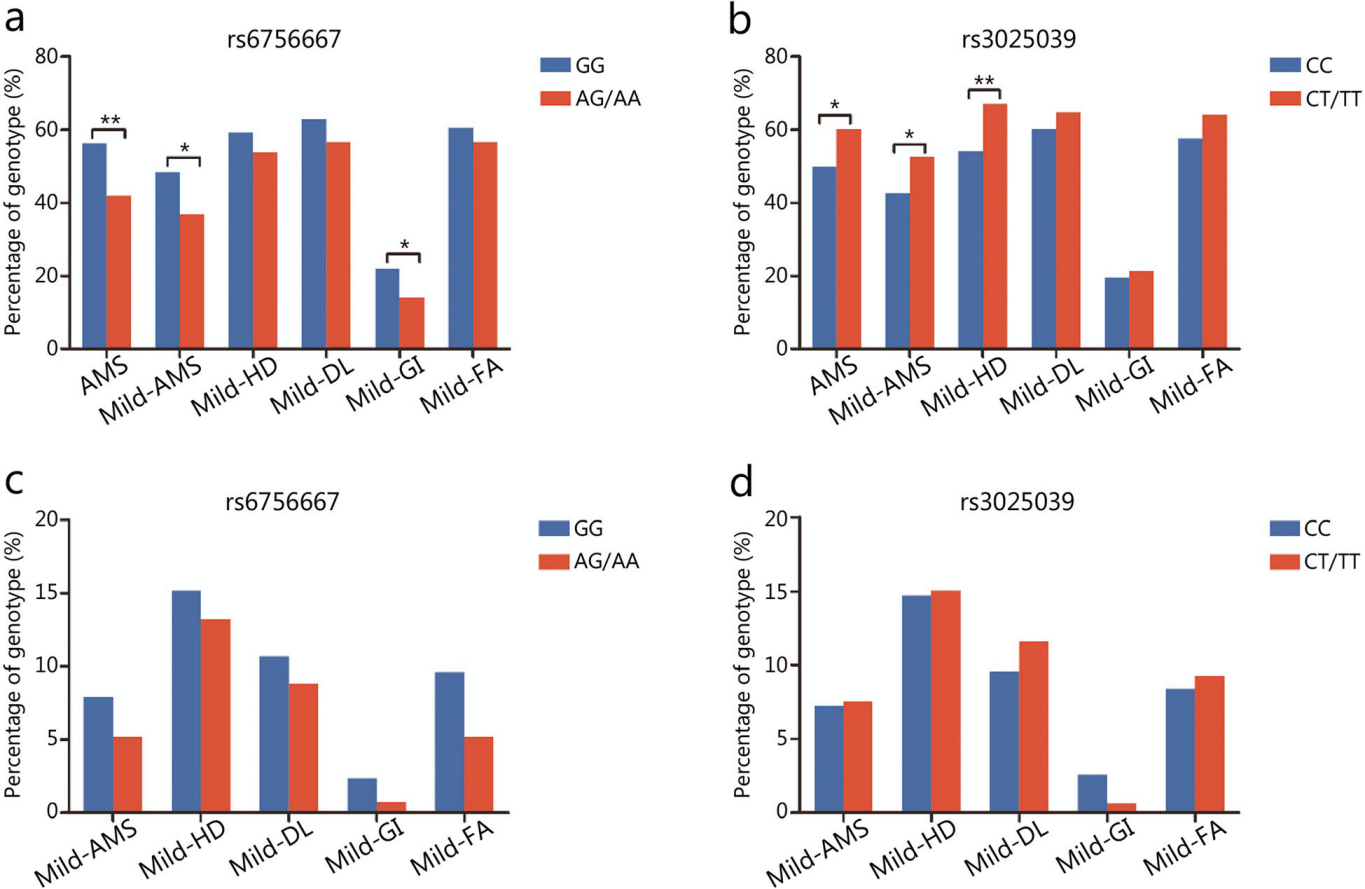
Associations between SNPs and the intensity of AMS/AMS-related symptoms. **a** and **b** exhibited the genotype frequencies of rs6756667 and rs3025039 associated with mild AMS/AMS-related symptoms, respectively. **c** and **d** exhibited the genotype frequencies of rs6756667 and rs3025039 associated with moderate-severe AMS/AMS-related symptoms, respectively. Blue. Wild-type homozygote; Red. Heterozygote type and variant-type homozygote. AMS. Acute mountain sickness; HD. Headache; DL. Dizziness and light-headedness; GI. Gastrointestinal symptoms; FA. Fatigue and/or weakness; MS, Moderate and severe groups. ^*^P < 0.05, ^**^P < 0.01

Association analyses between *EPAS1*-rs6756667, *VEGFA*-rs3025039 and the intensity of AMS/AMS-related symptoms were performed using the chi-square test. The rs6756667 GG genotype was associated with a significantly increased risk of AMS (*P* = 0.003), especially mild AMS (*P* = 0.018). Moreover, the rs6756667 GG genotype showed a significantly increased risk of mild GI symptoms (*P* = 0.040, Fig. 2a). The rs3025039 CT/TT genotype was associated with a significantly increased risk of AMS (*P* = 0.017), especially mild AMS (*P* = 0.019). In addition, the rs3025039 CT/TT genotype exhibited a significantly increased risk of mild headache (*P* = 0.003, Fig. 2b). However, no significant associations were found between SNPs and moderate-severe AMS/AMS-related symptoms (Fig. 2c-d).

### The combined effects of *EPAS1*-rs6756667 and *VEGFA*-rs3025039

To explore the combined effects of *EPAS1* and *VEGFA*, we divided the study subjects into 4 subgroups according to the rs6756667 GG and rs3025039 CT/TT status (Table 7). Subjects carrying both the rs6756667 GG and rs3025039 CC genotypes were more likely to develop AMS than those in subgroup 1 (*OR* = 2.06, 95% CI 1.30–3.27, *P* = 0.002). Subjects carrying both the rs6756667 GA/AA and rs3025039 CT/TT genotypes had an increased risk of AMS compared with those in subgroup 1 (*OR* = 2.35, 95% CI 1.07–5.17, *P* = 0.033). Moreover, compared with non-AMS subjects, AMS patients were more often carriers of both the rs6756667 GG genotype and the rs3025039 CT/TT genotype: 25.6% vs. 19.0% (*OR* = 2.70; 95% CI 1.59–4.61; *P* < 0.001). All results remained significant after adjusting for age, BMI and smoking status.

**Table 7:** Distribution of rs6756667 and rs3025039 in study subjects

Subgroup	Multiple SNPs		AMS group (*n* = 318)	Non-AMS group (*n* = 284)	*OR* (95% CI)	*P*-value	*OR* (95% CI)^a^	*P*-value^a^
	*EPAS1*-rs6756667	*VEGFA*-rs3025039						
1	GA/AA	CC	37 (11.7)	64 (22.7)	1		1	
2	GA/AA	CT/TT	20 (6.2)	15 (5.4)	2.31 (1.06–5.04)	0.036*	2.35 (1.07–5.17)	0.033*
3	GG	CC	177 (56.5)	151 (52.9)	2.03 (1.28–3.21)	0.003*	2.06 (1.30–3.27)	0.002*
4	GG	CT /TT	84 (25.6)	54 (19.0)	2.69 (1.58–4.57)	< 0.001*	2.70 (1.59–4.61)	< 0.001*

Subgroup 1: Subjects carrying neither rs6756667 “GG” genotype nor rs3025039 “CT/TT” genotype; Subgroup 2: Subjects carrying rs3025039 “CT/TT” genotype but no rs6756667 “GG” genotype; Subgroup 3: Subjects carrying rs6756667 “GG” genotype but no rs3025039 “CT/TT” genotype; Subgroup 4: Subjects carrying both rs3025039 “CT/TT” genotype and rs6756667 “GG” genotype. Two subjects were excluded because the genotyping results of *VEGFA*-rs3025039 were missing. ^a^ adjusted for age, BMI and smoking status; * *P* < 0.05 indicated significant difference. *SNP* Single nucleotide polymorphism; *AMS* Acute mountain sickness; *OR* Odds ratio; *CI* Confidence interval

## Discussion

In our present cross-sectional study, the contributions of 23 SNPs in 7 genes involved in sensing low oxygen levels to the risk of AMS were assessed in 604 young, healthy Han Chinese men who rapidly ascended to HA. Of them, we found that 4 SNPs (rs6756667 in *EPAS1*, rs3025039 in *VEGFA,* rs7292407 in *PPARA* and rs2153364 in *EGLN1*) were associated with the risk of developing AMS even though the results turned insignificant after FDR testing. Further stratified analysis revealed that AMS patients with the rs6756667 GG genotype had a higher risk of developing mild AMS and mild GI symptoms than those with other genotypes. In contrast, the rs3025039 CC genotype was associated with a lower risk of mild AMS and mild headache. These data indicate that *EPAS1* and *VEGFA* SNPs play important but different roles in the physiological effects of AMS in the Chinese Han population. To the best of our knowledge, this is the first epidemiological study to demonstrate such an association with AMS-related symptoms. The results of the current study provide novel information about *EPAS1* and *VEGFA* under conditions of acute hypobaric hypoxia, perhaps revealing a role for these genes in AMS; however, further study is required to confirm this hypothesis.

We showed 4 SNPs (rs6756667 in *EPAS1*, rs3025039 in *VEGFA,* rs7292407 in *PPARA* and rs2153364 in *EGLN1*) were associated with the risk of developing AMS. Comparing these 4 SNPs under dominant model, *EPAS1*-rs6756667 showed strongest association with the occurrence of AMS, while the other 3 SNPs showed relatively weak association (*P* = 0.003 vs. *P* > 0.01), which implicating at least in our tested gene SNPs, *EPAS1* variants might play more important role than other genes on HIF pathway. Furthermore, following the revised diagnosis criteria of LLSS, rs6756667 in *EPAS1* also showed positive association with AMS even without the influence of sleeping disturbance [18]. Although after FDR correction, only rs6756667 in *EPAS1* retained significance under codominant model. Our results need to be further validated in a larger sample size.

Although our results showed that rs6756667 in *EPAS1* correlates significantly with the development of GI symptoms, leading to a higher risk of AMS, the relationship may be difficult to interpret. *EPAS1* has 16 exons extending over 90 kb on chromosome 2, with a large first intron (50 kb) [26]. In our present study, all the selected *EPAS1* SNPs, rs13419896, rs4953354, rs6756667 and rs1868092, are located in *EPAS1* introns, which may affect binding to various transcription factors. The exonic SNPs were not selected because previous studies showed that the *EPAS1* SNPs with the greatest differences in frequency between Tibetans and non-Tibetan Chinese individuals are mainly located within introns [8, 27–29]. Hypoxia alters genome-wide gene expression at both the transcription and translation levels. Ribosome density at the 5′-UTR significantly increases under hypoxic conditions and is positively correlated with the presence of upstream open reading frames in the 5′-UTR of mRNAs [30]. Interestingly, in a study of native Tibetans residing at different HAs, the rs6756667 A allele was dominant. As the altitude increased, there was a linear decreasing trend in GG and AG carriers and an increasing trend in AA carriers [31]. Studies of Tibetans revealed that this population comprises a mixture of ancestral populations related to the Sherpa and Han Chinese [32]. Thus, it is reasonable to speculate that the different genetic backgrounds between lowlanders, such as Han Chinese, and highlanders, such as Tibetans, as related to *EPAS1* rs6756667 might be attributed to adaptation at HA. The rs6756667 A allele may be an allele that increases fitness to HA-related hypoxia. *EPAS1* was initially detected in endothelial cells, but expression has also been found in the lung, placenta, kidney, heart, liver, small intestine and many other organs associated with the regulation of oxygen metabolism [33, 34]. *EPAS1* was shown in genome-wide association studies to be associated with lower hemoglobin concentration and hypoxia adaptation in Tibetans [27, 35]. The adaptability of the Tibetan and plateau Han populations is mainly due to the strong affinity of hemoglobin for oxygen, which provides sufficient oxygen to tissues and organs [36]. *EPAS1* was also shown by whole-exome sequencing to be highly significantly associated with HA-related pulmonary hypertension in Angus cattle [37]. A recent study showed that HIF-1α and EPAS1/HIF-2α are upregulated in intestinal tissues from patients with GI vascular malformations [38]. VEGF is a direct target of EPAS1, and EPAS1 enhances vessel formation by endothelial cells through NOTCH1, Ang2 and DLL4 [39]. Iron and glucose homeostasis in the gut and liver may also be involved in regulating the GI system through the *EPAS1* gene under acute hypoxia [39, 40]. However, how *EPAS1,* and the intronic SNP in particular, affects GI symptoms remains largely unknown.

We also showed that *VEGFA* rs3025039 is associated with the risk of developing AMS, which is consistent with previous findings [19, 41]. Furthermore, we revealed that *VEGFA* rs3025039 is associated with headache, the most important symptom for the diagnosis of AMS. The SNP rs3025039 is a C/T single-nucleotide variation denoted as 936 C/T at the beginning of chromosome 6; the affected gene has a 14-kb coding region with 8 exons and 7 introns [42]. Unlike rs6756667 in *EPAS1*, relationships with the SNP rs3025039 and other *VEGFA* gene variants have been widely reported for HA illness and for cancer, heart disease, diabetes, neurological diseases and many other types of diseases. It is reasonable to assume that *VEGFA* gene variants may contribute to variations in VEGF serum levels and to altered susceptibility to diseases related to altered angiogenesis. In Bahraini [43] and southern Italian subjects [44], the TT genotype of rs3025039 was associated with lower VEGF levels. However, we could not find any reports on this topic in the Chinese Han population at sea level.

It remains to be determined whether plasma VEGF contributes to the pathogenesis of AMS. VEGF is a selective endothelial cell mitogen that induces vascular permeability and may be involved in increasing blood-brain barrier permeability in rather severe illness. The plasma levels of both VEGF and its soluble receptor (sFlt-1) are significantly increased in HAPE patients compared to non-HAPE controls [45]. Evidence regarding VEGF from AMS studies remains controversial. Absolute values or changes in VEGF were not correlated with AMS scores [46, 47]. On the other hand, Ding et al. reported that among Chinese Han subjects (*n* = 40), the AMS group had a higher VEGF plasma concentration than the control group at both sea level and HA [19]. In indirect agreement with the results of this previous study, we showed that individuals carrying rs3025039 CT/TT exhibited a higher frequency of developing AMS than those carrying the wild-type CC allele. We speculated that the same SNPs may not have the same contributions to protein production in different races and regions; in support this hypothesis, rs3025039 has different effects on VEGF plasma levels in populations from three villages in Southern Italy [44]. Further investigation is necessary to elucidate the relationship between *VEGFA* variants and VEGF serum levels and the underlying mechanisms in the Chinese Han population.

This is the first study to show that *VEGFA* rs3025039 is associated with headache in AMS. However, no direct evidence has been reported regarding the association of VEGF plasma levels with HA-related headache. In a rat model, recurrent headache induced by episodic inflammatory soup blood-brain barrier disruption was accompanied by increased VEGF expression [48]. Recently, Zhao et al. [49] reported that the rs3025039 TC/TT genotypes significantly increased the risk of poor recovery from ischemia and stroke in Chinese patients, which may indirectly explain why rs3025039 CT/TT carriers had a higher risk of headache and AMS in our study. Although we did not measure serum VEGF levels in our subjects, our results support the hypothesis that VEGF is an important component of the pathogenesis of AMS. The significant association of SNP rs3025039 with AMS needs to be validated in further studies with larger populations.

The present study does have certain limitations. First, not all tag SNPs in the HIF pathway were selected. Second, the number of subjects carrying the *EPAS1* rs6756667 AA genotype in this study was quite small (*n* = 6), and statistical errors may exist; more subjects must be included in future studies to verify the association between SNPs and AMS. Furthermore, our study was limited to young Chinese Han males, and therefore, the results may not be applicable to all Han populations, including individuals of other genders and ages.

## Conclusions

Despite the limited power of clinical association studies, the results using the novel LLSS standard have shown for the first time that variants in *EPAS1* and *VEGFA* are related to the susceptibility to AMS in the Chinese Han population. More importantly, *EPAS1* and *VEGFA* variants may affect the risk of AMS through different AMS-related symptoms. Our results provide novel evidence regarding the involvement of genetic factors in the development of AMS. These gene variants have potential utility in screening susceptible populations and predicting the clinical symptoms that lead to AMS, which will benefit soldiers and travelers who rapidly ascend to plateaus.

## Supplementary information

**Additional file 1. **Haplotype block maps for SNPs in *EGLN1*, *HIF1A*, *HIF1AN*, *PPARA*, and *VEGFA*.

**Additional file 2:Table S1.** Primer sequences for MALDI-TOF MS.

**Additional file 3:Table S2.** Basic SNP information among subjects.

**Additional file 4: Table S3.** Negative associations between SNP sites and AMS.

**Additional file 5: Table S4 .**Associations between SNPs and AMS related-headache.

**Additional file 6: Table S5.** Associations between SNPs and AMS related-dizziness/light-headedness.

**Additional file 7: Table S6.** Associations between SNPs and AMS related-gastrointestinal symptoms.

**Additional file 8: Table S7.** Associations between SNPs and AMS related-fatigue.

## Data Availability

All data generated or analyzes during this study are included in this published article and its supplementary information files.

## Abbreviations

AMS: Acute mountain sickness
BMI: Body mass index
BP: Blood pressure
D’: linkage disequilibrium coefficient
EPAS1: Endothelial PAS domain-containing protein 1
EGLN1: Egl nine homolog 1
FIH-1: Factor inhibiting HIF-1α
GI: Gastrointestinal symptoms
HA: High altitude
HACE: High-altitude cerebral edema
HAPE: High-altitude pulmonary edema
HD: Headache
HIF: Hypoxia-inducible factor
HIF1AN: Hypoxia-inducible factor 1-α inhibitor
HR: Heart rate
LD: Linkage disequilibrium
LLSS: Lake Louise scoring system
LOD: Logarithm of odds
*OR*: Odd ratio
PHD2: Prolyl hydroxylase domain protein 2
SaO_2_: Arterial oxygen saturation
SNP: Single nucleotide polymorphism
SpO_2_: Pulse oxygen saturation
VEGF: Vascular endothelial growth factor
