# Supplementary material for: EPAS1 and VEGFA gene variants are related to the symptoms of acute mountain sickness in Chinese Han population: a cross-sectional study
Source: Mil Med Res. 2020 Jul 27;7:35. doi: 10.1186/s40779-020-00264-6 (PMC7385974; doi:10.1186/s40779-020-00264-6)
Supplement: Supplementary file 1 — Additional file 1. Haplotype block maps for SNPs in EGLN1, HIF1A, HIF1AN, PPARA, and VEGFA. [file 40779_2020_264_MOESM1_ESM.docx]

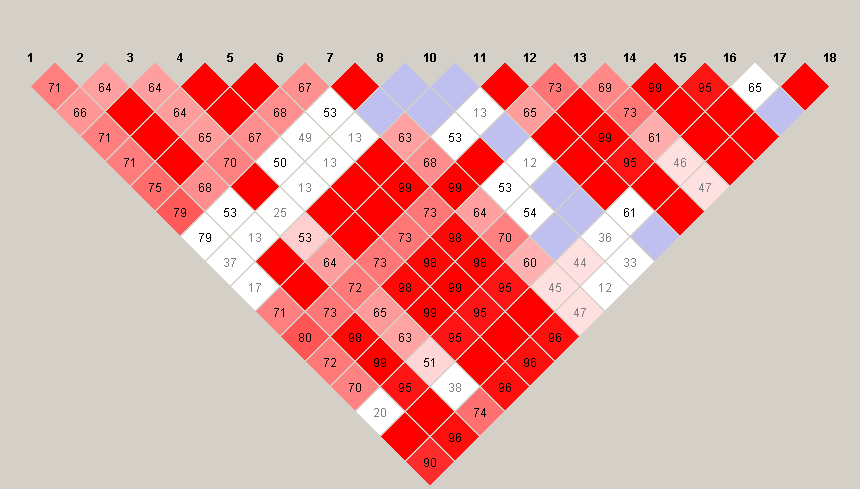


**Figure S1** Haplotype block map for SNPs in *EGLN1*

The corresponding SNP in Figure S1

| Number | SNP |
| --- | --- |
| 1 | rs1339891 |
| 2 | rs2066140 |
| 3 | rs2275279 |
| 4 | rs2486736 |
| 5 | rs2790882 |
| 6 | rs480902 |
| 7 | rs12406290 |
| 8 | rs12757362 |
| 9 | rs1339894 |
| 10 | rs1361384 |
| 11 | rs1538667 |
| 12 | rs2009873 |
| 13 | rs2153364 |
| 14 | rs2486729 |
| 15 | rs2739513 |
| 16 | rs2808609 |
| 17 | rs508618 |
| 18 | rs7542797 |

Linkage disequilibrium analyses between SNPs in *EGLN1*

| SNP | | D’ | LOD | *r*^2^ |
| --- | --- | --- | --- | --- |
| rs1339891 | rs7542797 | 0.9 | 75.82 | 0.622 |
| rs2066140 | rs2486736 | 1.0 | 261.67 | 1.0 |
| rs2066140 | rs2790882 | 1.0 | 263.49 | 1.0 |
| rs2066140 | rs480902 | 1.0 | 260.02 | 0.993 |
| rs2066140 | rs2009873 | 1.0 | 253.04 | 0.983 |
| rs2066140 | rs2486729 | 0.982 | 232.22 | 0.942 |
| rs2066140 | rs2739513 | 0.996 | 241.86 | 0.975 |
| rs12406290 | rs2153364 | 0.996 | 234.55 | 0.985 |
| rs1538667 | rs2808609 | 1.0 | 73.94 | 0.98 |
|  | |  |  |  |


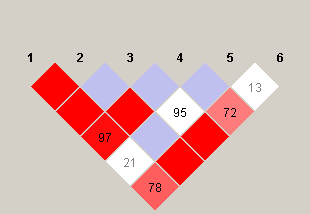


**Figure S2** Haplotype block map for tag SNPs in *HIF1A*

The corresponding SNP in Figure S2

| Number | | SNP | |
| --- | --- | --- | --- |
| 1 | rs12434438 | |  |
| 2 | rs2301104 | |  |
| 3 | rs2301112 | |  |
| 4 | rs966824 | |  |
| 5 | rs11549467 | |  |
| 6 | rs2301113 | |  |

Linkage disequilibrium analyses between SNPs in *HIF1A*

| SNP | | D’ | LOD | *r*^2^ |
| --- | --- | --- | --- | --- |
| rs12434438 | rs966824 | 0.973 | 107.52 | 0.661 |


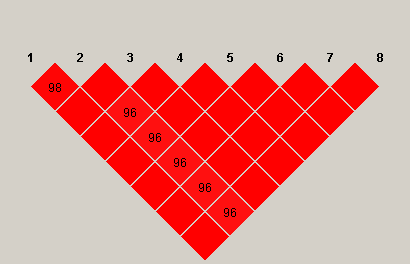


**Figure S3** Haplotype block map for tag SNPs in *HIF1AN*

The corresponding SNP in Figure S3

| Number | SNP |
| --- | --- |
| 1 | rs10883512 |
| 2 | rs11190602 |
| 3 | rs2295778 |
| 4 | rs1054399 |
| 5 | rs11190613 |
| 6 | rs11292 |
| 7 | rs11816840 |
| 8 | rs3750633 |

Linkage disequilibrium analyses between SNPs in *HIF1AN*

| SNP | | D’ | LOD | *r*^2^ |
| --- | --- | --- | --- | --- |
| rs10883512 | rs11190602 | 0.987 | 74.76 | 0.62 |
| rs10883512 | rs1054399 | 1.0 | 109.75 | 0.976 |
| rs10883512 | rs11190613 | 1.0 | 110.6 | 0.977 |
| rs10883512 | rs11292 | 1.0 | 110.6 | 0.977 |
| rs10883512 | rs11816840 | 1.0 | 110.6 | 0.977 |
| rs10883512 | rs3750633 | 1.0 | 109.75 | 0.976 |
|  | |  |  |  |


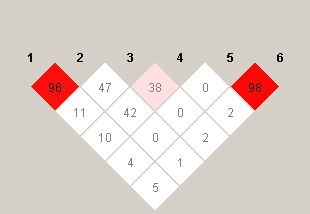


**Figure S4** Haplotype block map for tag SNPs in *PPARA*

The corresponding SNP in Figure S4

| Number | | SNP |
| --- | --- | --- |
| 1 | rs135538 | |
| 2 | rs4253623 | |
| 3 | rs4253681 | |
| 4 | rs4253747 | |
| 5 | rs6520015 | |
| 6 | rs7292407 | |

Linkage disequilibrium analyses between SNPs in *PPARA*

| SNP | | D’ | LOD | *r*^2^ |
| --- | --- | --- | --- | --- |
| rs6520015 | rs7292407 | 0.986 | 128.24 | 0.816 |


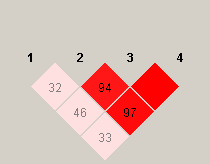


**Figure S5** Haplotype block map for tag SNPs in *VEGFA*

The corresponding SNP in Figure S5

| Number | | SNP |
| --- | --- | --- |
| 1 | rs1413711 | |
| 2 | rs3025039 | |
| 5 | rs10434 | |
| 6 | rs3025040 | |

Linkage disequilibrium analyses between SNPs in *VEGFA*

| SNP | | D’ | LOD | *r*^2^ |
| --- | --- | --- | --- | --- |
| rs3025039 | rs3025040 | 0.974 | 153.86 | 0.943 |

D’. Linkage disequilibrium coefficient; LD. Linkage disequilibrium; LOD. Logarithm of odds; SNP. Single nucleotide polymorphism.
