# Supplementary material for: EPAS1 and VEGFA gene variants are related to the symptoms of acute mountain sickness in Chinese Han population: a cross-sectional study
Source: Mil Med Res. 2020 Jul 27;7:35. doi: 10.1186/s40779-020-00264-6 (PMC7385974; doi:10.1186/s40779-020-00264-6)
Supplement: Supplementary file 2 — Additional file 2:Table S1. Primer sequences for MALDI-TOF MS. [file 40779_2020_264_MOESM2_ESM.docx]

**Table S1** Primer sequences for MALDI-TOF MS

| SNPs | Gene | Forward primer (5’ to 3’) | Reverse primer (5’ to 3’) | Sequencing primer (5’ to 3’) |
| --- | --- | --- | --- | --- |
| rs2066140 | *EGLN1* | ACGTTGGATGAGCGTTTATACATTTTC CCC | ACGTTGGATGAGTGAGGCATCTCTA GTTTA | TTATACATTTTCCCCATTATTTGTAT |
| rs508618 | *EGLN1* | ACGTTGGATGATCTTTTGGCCTTGAGA TCC | ACGTTGGATGGTCTATAGCAAGGGT ATTTG | GCCCCTCATTGGCTTCT |
| rs1538667 | *EGLN1* | ACGTTGGATGTTTGATCTCGAACTCCT CGG | ACGTTGGATGTAACACTACTCACTTC ACAG | CTGTGGTTACAGACTTGA |
| rs2153364 | *EGLN1* | ACGTTGGATGCCATCAAATAGAACACT TGC | ACGTTGGATGCCTGAAATCAAGGAT CTTTGG | cCTTGCAACACTTTAATGAATAC |
| rs1361384 | *EGLN1* | ACGTTGGATGGCCTGAATAGGTGAAA ATCC | ACGTTGGATGCCATCAAATAGAACAC TTGC | GGAACTATTTGAAATCAGTATTT |
| rs1339894 | *EGLN1* | ACGTTGGATGGCTGTTATGTGCCCAAT CTG | ACGTTGGATGGTACCCACTTCATAG CATTG | GCCCAATCTGTGTTCTAA |
| rs12757362 | *EGLN1* | ACGTTGGATGGGTAGTTGCCGTGTAT TTCC | ACGTTGGATGCCCATGAGCAATATTT CGTC | ggagaACATCATGAGGCACATATGTCT |
| rs13419896 | *EPAS1* | ACGTTGGATGCTGAACCAGAGTCAGT AACC | ACGTTGGATGAACCCTTCCTGGTTG AGTAG | GAAAAGAGTCAGTAACCAATCCTAG |
| rs4953354 | *EPAS1* | ACGTTGGATGTCTGCCATTGTCTTGCA TTG | ACGTTGGATGAAGAGGCGAAATGTG CAGAC | TGAAAGTATTTATGGAGCATATCT |
| rs6756667 | *EPAS1* | ACGTTGGATGTCGGAATCGACAGACT GGTG | ACGTTGGATGCTGACCAAGAGTTGA TGCTG | AAACTGCTGTAAGGTGA |
| rs1413711 | *VEGFA* | TGACAATATTCTCCCGGGACC | AGTGTGACCTTCAGAGGCCC | CTTCCAAGGCCAGGGGGCA |
| rs3025039 | *VEGFA* | ACGTTGGATGAGACTCCGGCGGAAGC ATT | ACGTTGGATGCTCGGTGATTTAGCA GCAAG | GGGCGGGTGACCCAGCA |
| rs10434 | *VEGFA* | ACGTTGGATGATGGGCTGCTTCTTCC AACA | ACGTTGGATGATGGGCTGCTTCTTC CAACA | GACATCTGCCAGTGG |
| rs4253623 | *PPARA* | CGTTGGATGAGCATGAATCCATGAAA CTG | ACGTTGGATGCCGGCAAAAATAGGT TCTTC | GACCAGAATATAAAAAAGAAACTTAAAG |
| rs135538 | *PPARA* | ACGTTGGATGGTGCCTTTCCCTCATTA GTC | ACGTTGGATGATTACAGCCACTCTTG TGGG | AAGTTCATAGACATCTCTCTT |
| rs4253681 | *PPARA* | ACGTTGGATGAGCCTGGGAGGTCGAT ATTG | ACGTTGGATGTTGGACACAGAGTTT CCTTC | GTGAGCTGTAATTGCACCA |
| rs4253747 | *PPARA* | ACGTTGGATGCCCAAAGCAAAAATGA ACTCC | ACGTTGGATGATCATGGCCCTAAGG AAAAG | GCCCCAAGCAAAAATGAACTCCTTCGCA |
| rs1680710 | *EGLN3* | ACGTTGGATGCAGACGAGAACTCAAA TAGC | ACGTTGGATGATGAGCTCACCCTTTA ACAC | tccaaTTCCTTTTTTAAAATACATCACC |
| rs11156819 | *EGLN3* | ACGTTGGATGTCTCTGTGGCATCTACC ACC | ACGTTGGATGAGCGAGGGAATGAAC CTTAC | CCACCTCCATTTTTTTCTTTTCAAA |
| rs2301104 | *HIF1A* | ACGTTGGATGTCAAGCTCACAAGTGA AGGG | ACGTTGGATGCCTTTTGTTTGAGGAA GCAG | GGATCAGAGGTATCTGCCTCTAGATC |
| rs12434438 | *HIF1A* | ACGTTGGATGGGAGACTTATGACTAG AGAC | ACGTTGGATGCCTGCACCATGTTAA GCATT | AGACAAAGGAAAGGCAAAAC |
| rs2301112 | *HIF1A* | ACGTTGGATGCCCTACTCTTAGCTTGT CAG | ACGTTGGATGATAACTTGATAAATGA GGC | ACCTAATGTAGAATTATTTACCAC |
| rs2301113 | *HIF1A* | ACGTTGGATGCCTTTGAACTGAGAAG GCAC | ACGTTGGATGTTAGGTTTCTACTCCC ACCC | GGGGTAGAAGGCACACTCCT |
| rs11549467 | *HIF1A* | ACGTTGGATGTTGAGGACTTGCGCTTT CAG | ACGTTGGATGCTTCCAGTTACGTTCC TTCG | CGCTTTCAGGGCTTG |

MALDI-TOF MS. Matrix-Assisted Laser Desorption Ionization-Time of Flight Mass Spectrometer; SNP. Single nucleotide polymorphism.
