# Supplementary material for: EPAS1 and VEGFA gene variants are related to the symptoms of acute mountain sickness in Chinese Han population: a cross-sectional study
Source: Mil Med Res. 2020 Jul 27;7:35. doi: 10.1186/s40779-020-00264-6 (PMC7385974; doi:10.1186/s40779-020-00264-6)
Supplement: Supplementary file 3 — Additional file 3:Table S2. Basic SNP information among subjects. [file 40779_2020_264_MOESM3_ESM.docx]

**Table S2** Basic SNP information among subjects

| SNP ID | Allele | Gene name | Chromosome position^#^ | N | MAF^a^ (%) | MAF^b^ (%) | Wild type [*n* (%)] | Heterozygote [*n* (%)] | Mutant [*n* (%)] | HWE *P*-value |
| --- | --- | --- | --- | --- | --- | --- | --- | --- | --- | --- |
| rs2066140 | G/C | *EGLN1* | Chr1:231368565 | 601 | 42.0 | 44.2 | 202 (33.6) | 292 (48.6) | 107 (17.8) | 0.930 |
| rs508618 | A/G | *EGLN1* | Chr1:231396566 | 597 | 11.0 | 11.2 | 472 (79.1) | 122 (20.4) | 3 (0.5) | 0.130 |
| rs1538667 | A/G | *EGLN1* | Chr1:231418597 | 599 | 4.0 | 3.9 | 550 (91.8) | 48 (8.0) | 1 (0.2) | 1.000 |
| rs1339891 | G/A | *EGLN1* | Chr1:231420649 | 603 | 10.0 | 11.2 | 489 (81.1) | 109 (18.1) | 5 (0.8) | 0.820 |
| rs2153364 | A/G | *EGLN1* | Chr1:231424474 | 549 | 48.0 | 49.0 | 153 (27.9) | 267 (48.6) | 129 (23.5) | 0.550 |
| rs1361384 | A/G | *EGLN1* | Chr1:231424487 | 599 | 0.0 | 0.0 | 597 (99.7) | 2 (0.3) | 0 (0.0) | 1.000 |
| rs1339894 | G/A | *EGLN1* | Chr1:231424811 | 587 | 0.0 | 0.0 | 586 (99.8) | 1 (0.2) | 0 (0.0) | 1.000 |
| rs12757362 | G/C | *EGLN1* | Chr1:231426746 | 599 | 3.0 | 2.9 | 561 (93.7) | 38 (6.3) | 0 (0.0) | 1.000 |
| rs2275279 | A/T | *EGLN1* | Chr1:231591348 | 603 | 27.0 | 30.6 | 324 (53.7) | 229 (38.0) | 50 (8.3) | 0.300 |
| rs13419896 | G/A | *EPAS1* | Chr2:46329206 | 601 | 31.0 | 30.6 | 284 (47.2) | 257 (42.8) | 60 (10.0) | 0.850 |
| rs4953354 | A/G | *EPAS1* | Chr2:46348249 | 603 | 13.0 | 11.7 | 453 (75.1) | 140 (23.2) | 10 (1.7) | 1.000 |
| rs6756667 | G/A | *EPAS1* | Chr2:46352270 | 604 | 12.0 | 13.6 | 468 (77.5) | 130 (21.5) | 6 (1.0) | 0.440 |
| rs1868092 | G/A | *EPAS1* | Chr2:46387063 | 604 | 8.0 | 8.3 | 512 (84.8) | 84 (13.9) | 8 (1.3) | 0.054 |
| rs1413711 | G/A | *VEGFA* | Chr6:43772941 | 589 | 27.0 | 27.2 | 306 (51.9) | 252 (42.8) | 31 (5.3) | 0.027*** |
| rs3025039 | C/T | *VEGFA* | Chr6:43784799 | 602 | 15.0 | 18.5 | 429 (71.3) | 162 (26.9) | 11 (1.3) | 0.430 |
| rs10434 | G/A | *VEGFA* | Chr6:43785475 | 593 | 24.0 | 20.9 | 336 (56.7) | 226 (38.1) | 31 (5.2) | 0.430 |
| rs7292407 | C/A | *PPARA* | Chr22:46057832 | 571 | 15.0 | 17.5 | 417 (73.0) | 135 (23.6) | 19 (3.3) | 0.071 |
| rs4253623 | A/G | *PPARA* | Chr22:46154203 | 597 | 14.0 | 13.6 | 445 (74.5) | 142 (23.8) | 10 (1.7) | 0.860 |
| rs135538 | G/C | *PPARA* | Chr22:46168728 | 601 | 44.0 | 48.1 | 194 (32.3) | 280 (46.6) | 127 (21.1) | 0.160 |
| rs4253681 | T/C | *PPARA* | Chr22:46183703 | 603 | 20.0 | 21.8 | 386 (64.0) | 196 (32.5) | 21 (3.5) | 0.610 |
| rs4253747 | T/A | *PPARA* | Chr22:46217340 | 603 | 21.0 | 19.9 | 377 (62.5) | 199 (33.0) | 27 (4.5) | 0.900 |
| rs1680710 | G/A | *EGLN3* | Chr14:33924897 | 595 | 4.0 | 4.4 | 547 (91.9) | 47 (7.9) | 1 (0.2) | 1.000 |
| rs11156819 | C/T | *EGLN3* | Chr14:33925527 | 596 | 29.0 | 29.6 | 302 (50.7) | 244 (40.9) | 50 (8.4) | 0.920 |
| rs2301104 | G/C | *HIF1A* | Chr14:61698310 | 603 | 6.0 | 9.7 | 526 (87.2) | 76 (12.6) | 1 (0.2) | 0.500 |
| rs12434438 | A/G | *HIF1A* | Chr14:61730580 | 587 | 24.0 | 24.8 | 336 (57.2) | 224 (38.2) | 27 (4.6) | 0.210 |
| rs2301112 | A/C | *HIF1A* | Chr14:61739455 | 564 | 5.0 | 5.3 | 511 (90.6) | 52 (9.2) | 1 (0.2) | 1.000 |
| rs2301113 | A/C | *HIF1A* | Chr14:61739830 | 595 | 33.0 | 33.0 | 259 (43.5) | 275 (46.2) | 61 (10.3) | 0.360 |
| rs11549467 | G/A | *HIF1A* | Chr14:61740857 | 599 | 4.0 | 2.9 | 557 (93.0) | 41 (6.8) | 1 (0.2) | 0.540 |
| rs2295778 | C/G | *HIF1AN* | Chr10:100536079 | 602 | 24.0 | 23.3 | 347 (57.6) | 221 (36.7) | 34 (5.6) | 1.000 |
| rs10883512 | A/G | *HIF1AN* | Chr10:100548759 | 603 | 8.0 | 8.7 | 516 (85.6) | 83 (13.8) | 4 (0.7) | 0.770 |

* *P*<0.05 indicated that the distribution of SNP in subjects did not fit Hardy-Weinberg equilibrium; ^#^ the reference panel for identifying chromosomal positions of SNPs was Genome Reference Consortium Human Build 38 path 12 (GRCh38.p12); ^a^ MAF of SNPs in 604 subjects; ^b^ MAF of SNPs in Han Chinese (Beijing, China). N. The number of samples of which SNPs were successfully detected among 604 subjects; SNP. Single nucleotide polymorphism; MAF. Minor allele frequency; HWE. Hardy-Weinberg equilibrium.
