# Supplementary material for: EPAS1 and VEGFA gene variants are related to the symptoms of acute mountain sickness in Chinese Han population: a cross-sectional study
Source: Mil Med Res. 2020 Jul 27;7:35. doi: 10.1186/s40779-020-00264-6 (PMC7385974; doi:10.1186/s40779-020-00264-6)
Supplement: Supplementary file 4 — Additional file 4: Table S3. Negative associations between SNP sites and AMS. [file 40779_2020_264_MOESM4_ESM.docx]

**Table S3** Negative associations between SNP sites and AMS

| SNP ID | Gene | Model | Allele/Genotype | AMS group (*n* = 320) | Non-AMS group (*n* = 284) | *OR* (95% CI) | *P*-value | *OR* (95% CI)^a^ | *P*-value^a^ | *Q*-value |
| --- | --- | --- | --- | --- | --- | --- | --- | --- | --- | --- |
| rs2066140 | *EGLN1* | Allele | G | 358 (56.1) | 338 (59.9) | - | 0.181 | - | - | - |
|  |  |  | C | 280 (43.9) | 226 (40.1) | - |  | - |  |  |
|  |  | Codominant | GG | 98 (30.7) | 104 (36.9) | 1 | 0.280 | 1 | 0.260 | 0.598 |
|  |  |  | CG | 162 (50.8) | 130 (46.1) | 1.32 (0.92-1.89) |  | 1.34 (0.93-1.93) |  |  |
|  |  |  | CC | 59 (18.5) | 48 (17.0) | 1.30 (0.81-2.09) |  | 1.28 (0.80-2.06) |  |  |
|  |  | Dominant | GG | 98 (30.7) | 104 (36.9) | 1 | 0.110 | 1 | 0.110 | 0.506 |
|  |  |  | CG/CC | 221 (69.3) | 178 (63.1) | 1.32 (0.94-1.85) |  | 1.33 (0.94-1.86) |  |  |
|  |  | Recessive | GG/CG | 260 (81.5) | 234 (83.0) | 1 | 0.640 | 1 | 0.730 | 0.840 |
|  |  |  | CC | 59 (18.5) | 48 (17.0) | 1.11 (0.73-1.68) |  | 1.08 (0.71-1.64) |  |  |
| rs508618 | *EGLN1* | Allele | A | 567 (89.4) | 499 (89.1) | - | 0.856 | - | - | - |
|  |  |  | G | 67 (10.6) | 61 (10.9) | - |  | - |  |  |
|  |  | Codominant | AA | 253 (79.8) | 219 (78.2) | 1 | 0.120 | 1 | 0.110 | 0.361 |
|  |  |  | AG | 61 (19.2) | 61 (21.8) | 0.87 (0.58-1.29) |  | 0.87 (0.58-1.30) |  |  |
|  |  |  | GG | 3 (1.0) | 0 (0.0) | NA (0.00-NA) |  | NA (0.00-NA) |  |  |
|  |  | Dominant | AA | 253 (79.8) | 219 (78.2) | 1 | 0.630 | 1 | 0.660 | 0.949 |
|  |  |  | AG/GG | 64 (20.2) | 61 (21.8) | 0.91 (0.61-1.35) |  | 0.92 (0.62-1.36) |  |  |
|  |  | Recessive | AA/AG | 314 (99.0) | 280 (100.0) | 1 | 0.051 | 1 | 0.045 | 0.259 |
|  |  |  | GG | 3 (1.0) | 0 (0.0) | NA (0.00-NA) |  | NA (0.00-NA) |  |  |
| rs1339891 | *EGLN1* | Allele | G | 569 (89.2) | 518 (91.2) | - | 0.242 | - | - | - |
|  |  |  | A | 69 (10.8) | 50 (8.8) | - |  | - |  |  |
|  |  | Codominant | GG | 253 (79.3) | 236 (83.1) | 1 | 0.490 | 1 | 0.520 | 0.748 |
|  |  |  | AG | 63 (19.8) | 46 (16.2) | 1.28 (0.84-1.94) |  | 1.27 (0.84-1.94) |  |  |
|  |  |  | AA | 3 (0.9) | 2 (0.7) | 1.40 (0.23-8.45) |  | 1.30 (0.21-7.96) |  |  |
|  |  | Dominant | GG | 253 (79.3) | 236 (83.1) | 1 | 0.230 | 1 | 0.250 | 0.821 |
|  |  |  | AG/AA | 66 (20.7) | 48 (16.9) | 1.28 (0.85-1.94) |  | 1.27 (0.84-1.93) |  |  |
|  |  | Recessive | GG/AG | 316 (99.1) | 282 (99.3) | 1 | 0.750 | 1 | 0.810 | 0.847 |
|  |  |  | AA | 3 (0.9) | 2 (0.7) | 1.34 (0.22-8.07) |  | 1.25 (0.20-7.64) |  |  |
| rs2275279 | *EGLN1* | Allele | A | 462 (72.4) | 415 (73.1) | - | 0.800 | - | - | - |
|  |  |  | T | 176 (27.6) | 153 (26.9) | - |  | - |  |  |
|  |  | Codominant | AA | 166 (52.0) | 158 (55.6) | 1 | 0.260 | 1 | 0.230 | 0.588 |
|  |  |  | AT | 130 (40.8) | 99 (34.9) | 1.25 (0.89-1.76) |  | 1.27 (0.90-1.79) |  |  |
|  |  |  | TT | 23 (7.2) | 27 (9.5) | 0.81 (0.45-1.47) |  | 0.81 (0.44-1.48) |  |  |
|  |  | Dominant | AA | 166 (52.0) | 158 (55.6) | 1 | 0.380 | 1 | 0.340 | 0.652 |
|  |  |  | AT/TT | 153 (48.0) | 126 (44.4) | 1.16 (0.84-1.59) |  | 1.17 (0.85-1.62) |  |  |
|  |  | Recessive | AA/AT | 296 (92.8) | 257 (90.5) | 1 | 0.310 | 1 | 0.290 | 0.556 |
|  |  |  | TT | 23 (7.2) | 27 (9.5) | 0.74 (0.41-1.32) |  | 0.73 (0.41-1.31) |  |  |
| rs13419896 | *EGLN1* | Allele | G | 446 (70.1) | 379 (70.0) | - | 0.238 | - | - | - |
|  |  |  | A | 190 (29.9) | 187 (30.0) | - |  | - |  |  |
|  |  | Codominant | GG | 157 (49.4) | 127 (44.9) | 1 | 0.500 | 1 | 0.570 | 0.690 |
|  |  |  | GA | 132 (41.5) | 125 (44.2) | 0.85 (0.61-1.20) |  | 0.86 (0.61-1.21) |  |  |
|  |  |  | AA | 29 (9.1) | 31 (10.9) | 0.76 (0.43-1.32) |  | 0.79 (0.45-1.38) |  |  |
|  |  | Dominant | GG | 157 (49.4) | 127 (44.9) | 1 | 0.270 | 1 | 0.310 | 0.792 |
|  |  |  | GA/AA | 161 (50.6) | 158 (55.1) | 0.83 (0.61-1.15) |  | 0.85 (0.61-1.17) |  |  |
|  |  | Recessive | GG/GA | 289 (90.9) | 252 (89.0) | 1 | 0.450 | 1 | 0.530 | 0.717 |
|  |  |  | AA | 29 (9.1) | 31 (10.9) | 0.82 (0.48-1.39) |  | 0.84 (0.49-1.44) |  |  |
| rs4953354 | *EPAS1* | Allele | A | 558 (87.2) | 488 (86.2) | - | 0.621 | - | - | - |
|  |  |  | G | 82 (12.8) | 78 (13.8) | - |  | - |  |  |
|  |  | Codominant | AA | 242 (75.6) | 211 (74.6) | 1 | 0.700 | 1 | 0.810 | 0.887 |
|  |  |  | AG | 74 (23.1) | 66 (23.3) | 0.98 (0.67-1.43) |  | 0.98 (0.67-1.43) |  |  |
|  |  |  | GG | 4 (1.2) | 6 (2.1) | 0.58 (0.16-2.09) |  | 0.66 (0.18-2.39) |  |  |
|  |  | Dominant | AA | 242 (75.6) | 211 (74.6) | 1 | 0.760 | 1 | 0.790 | 1.009 |
|  |  |  | AG/GG | 78 (24.3) | 72 (25.4) | 0.94 (0.65-1.37) |  | 0.95 (0.65-1.38) |  |  |
|  |  | Recessive | AA/AG | 316 (98.8) | 277 (97.9) | 1 | 0.400 | 1 | 0.530 | 0.677 |
|  |  |  | GG | 4 (1.2) | 6 (2.1) | 0.58 (0.16-2.09) |  | 0.66 (0.18-2.40) |  |  |
| rs1868092 | *EPAS1* | Allele | G | 593 (92.7) | 515 (90.7) | - | 0.211 | - | - | - |
|  |  |  | A | 47 (7.3) | 53 (9.3) | - |  | - |  |  |
|  |  | Codominant | GG | 276 (86.3) | 236 (83.1) | 1 | 0.460 | 1 | 0.550 | 0.744 |
|  |  |  | GA | 41 (12.8) | 43 (15.1) | 0.82 (0.51-1.29) |  | 0.83 (0.52-1.31) |  |  |
|  |  |  | AA | 3 (0.9) | 5 (1.8) | 0.51 (0.12-2.17) |  | 0.57 (0.13-2.43) |  |  |
|  |  | Dominant | GG | 276 (86.3) | 236 (83.1) | 1 | 0.280 | 1 | 0.330 | 0.690 |
|  |  |  | GA/AA | 44 (13.7) | 48 (16.9) | 0.78 (0.50-1.22) |  | 0.80 (0.51-1.25) |  |  |
|  |  | Recessive | GG/GA | 317 (99.1) | 279 (98.2) | 1 | 0.380 | 1 | 0.460 | 0.705 |
|  |  |  | AA | 3 (0.9) | 5 (1.8) | 0.53 (0.13-2.23) |  | 0.59 (0.14-2.49) |  |  |
| rs10434 | *VEGFA* | Allele | G | 479 (76.3) | 419 (75.1) | - | 0.635 | - | - | - |
|  |  |  | A | 149 (13.7) | 139 (24.9) | - |  | - |  |  |
|  |  | Codominant | GG | 181 (57.6) | 155 (55.6) | 1 | 0.880 | 1 | 0.880 | 0.920 |
|  |  |  | GA | 117 (37.3) | 109 (39.1) | 0.92 (0.66-1.29) |  | 0.92 (0.65-1.29) |  |  |
|  |  |  | AA | 16 (5.1) | 15 (5.3) | 0.91 (0.44-1.91) |  | 0.92 (0.44-1.93) |  |  |
|  |  | Dominant | GG | 181 (57.6) | 155 (55.6) | 1 | 0.610 | 1 | 0.610 | 1.002 |
|  |  |  | GA/AA | 133 (42.4) | 124 (44.4) | 0.92 (0.66-1.27) |  | 0.92 (0.66-1.27) |  |  |
|  |  | Recessive | GG/AG | 298 (94.9) | 264 (94.7) | 1 | 0.880 | 1 | 0.900 | 0.900 |
|  |  |  | AA | 16 (5.1) | 15 (5.3) | 0.94 (0.46-1.95) |  | 0.95 (0.46-1.98) |  |  |
| rs11156819 | *EGLN3* | Allele | C | 448 (70.9) | 400 (71.4) | - | 0.837 | - | - | - |
|  |  |  | T | 184 (29.1) | 160 (28.6) | - |  | - |  |  |
|  |  | Codominant | CC | 164 (51.9) | 138 (49.3) | 1 | 0.130 | 1 | 0.100 | 0.383 |
|  |  |  | CT | 120 (38.0) | 124 (44.3) | 0.81 (0.58-1.14) |  | 0.79 (0.56-1.12) |  |  |
|  |  |  | TT | 32 (10.1) | 18 (6.4) | 1.50 (0.80-2.78) |  | 1.49 (0.80-2.78) |  |  |
|  |  | Dominant | CC | 164 (51.9) | 138 (49.3) | 1 | 0.520 | 1 | 0.440 | 0.778 |
|  |  |  | CT/TT | 152 (48.1) | 142 (50.7) | 0.90 (0.65-1.24) |  | 0.88 (0.63-1.22) |  |  |
|  |  | Recessive | CC/CT | 284 (89.9) | 262 (93.6) | 1 | 0.100 | 1 | 0.098 | 0.322 |
|  |  |  | TT | 32 (10.1) | 18 (6.4) | 1.64 (0.90-2.99) |  | 1.65 (0.90-3.03) |  |  |
| rs2301104 | *HIF1A* | Allele | G | 597 (93.6) | 531 (93.5) | - | 0.951 | - | - | - |
|  |  |  | C | 41 (6.4) | 37 (6.5) | - |  | - |  |  |
|  |  | Codominant | GG | 278 (87.2) | 248 (87.3) | 1 | 0.460 | 1 | 0.430 | 0.706 |
|  |  |  | CG | 41 (12.8) | 35 (12.3) | 1.05 (0.65-1.69) |  | 1.04 (0.64-1.68) |  |  |
|  |  |  | CC | 0 (0.0) | 1 (0.4) | 0.00 (0.00-NA) |  | 0.00 (0.00-NA) |  |  |
|  |  | Dominant | GG | 278 (87.2) | 248 (87.3) | 1 | 0.950 | 1 | 0.980 | 1.025 |
|  |  |  | CG/CC | 41 (12.8) | 36 (12.7) | 1.02 (0.63-1.64) |  | 1.01 (0.62-1.63) |  |  |
|  |  | Recessive | GG/CG | 319 (100.0) | 283 (99.7) | 1 | 0.220 | 1 | 0.200 | 0.511 |
|  |  |  | CC | 0 (0.0) | 1 (0.4) | 0.00 (0.00-NA) |  | 0.00 (0.00-NA) |  |  |
| rs12434438 | *HIF1A* | Allele | A | 477 (76.7) | 419 (75.9) | - | 0.753 | - | - | - |
|  |  |  | G | 145 (23.3) | 133 (24.1) | - |  | - |  |  |
|  |  | Codominant | AA | 177 (56.9) | 159 (57.6) | 1 | 0.370 | 1 | 0.360 | 0.690 |
|  |  |  | AG | 123 (39.6) | 101 (36.6) | 1.09 (0.78-1.54) |  | 1.13 (0.80-1.59) |  |  |
|  |  |  | GG | 11 (3.5) | 16 (5.8) | 0.62 (0.28-1.37) |  | 0.64 (0.29-1.42) |  |  |
|  |  | Dominant | AA | 177 (56.9) | 159 (57.6) | 1 | 0.860 | 1 | 0.730 | 0.988 |
|  |  |  | AG/GG | 134 (43.1) | 117 (42.4) | 1.03 (0.74-1.43) |  | 1.06 (0.76-1.47) |  |  |
|  |  | Recessive | AA/AG | 300 (96.5) | 260 (94.2) | 1 | 0.190 | 1 | 0.210 | 0.483 |
|  |  |  | GG | 11 (3.5) | 16 (5.8) | 0.60 (0.27-1.31) |  | 0.61 (0.28-1.33) |  |  |
| rs2301112 | *HIF1A* | Allele | A | 564 (95.3) | 510 (95.1) | - | 0.924 | - | - | - |
|  |  |  | C | 28 (4.7) | 26 (4.9) | - |  | - |  |  |
|  |  | Codominant | AA | 269 (90.9) | 242 (90.3) | 1 | 0.490 | 1 | 0.550 | 0.703 |
|  |  |  | AC | 26 (8.8) | 26 (9.7) | 0.90 (0.51-1.59) |  | 0.90 (0.51-1.60) |  |  |
|  |  |  | CC | 1 (0.3) | 0 (0.0) | NA (0.00-NA) |  | NA (0.00-NA) |  |  |
|  |  | Dominant | AA | 269 (90.9) | 242 (90.3) | 1 | 0.810 | 1 | 0.810 | 0.981 |
|  |  |  | AC/CC | 27 (9.1) | 26 (9.7) | 0.93 (0.53-1.65) |  | 0.93 (0.53-1.64) |  |  |
|  |  | Recessive | AA/AC | 295 (99.7) | 268 (100.0) | 1 | 0.260 | 1 | 0.300 | 0.493 |
|  |  |  | CC | 1 (0.3) | 0 (0.0) | NA (0.00-NA) |  | NA (0.00-NA) |  |  |
| rs2301113 | *HIF1A* | Allele | A | 431 (68.4) | 362 (64.6) | - | 0.169 | - | - | - |
|  |  |  | C | 199 (31.6) | 198 (35.4) | - |  | - |  |  |
|  |  | Codominant | AA | 139 (44.1) | 120 (42.9) | 1 | 0.038* | 1 | 0.051 | 0.391 |
|  |  |  | AC | 153 (48.6) | 122 (43.6) | 1.08 (0.77-1.52) |  | 1.09 (0.77-1.53) |  |  |
|  |  |  | CC | 23 (7.3) | 38 (13.6) | 0.52 (0.29-0.93) |  | 0.54 (0.30-0.96) |  |  |
|  |  | Dominant | AA | 139 (44.1) | 120 (42.9) | 1 | 0.760 | 1 | 0.820 | 0.898 |
|  |  |  | AC/CC | 176 (55.9) | 160 (57.1) | 0.95 (0.69-1.31) |  | 0.96 (0.69-1.33) |  |  |
|  |  | Recessive | AA/AC | 292 (92.7) | 242 (86.4) | 1 | 0.012* | 1 | 0.017^*^ | 0.130 |
|  |  |  | CC | 23 (7.3) | 38 (13.6) | 0.50 (0.29-0.87) |  | 0.52 (0.30-0.90) |  |  |
| rs2295778 | *HIF1AN* | Allele | C | 486 (76.2) | 429 (75.8) | - | 0.877 | - | - | - |
|  |  |  | G | 152 (23.8) | 137 (24.2) | - |  | - |  |  |
|  |  | Codominant | CC | 184 (57.7) | 163 (57.6) | 1 | 0.930 | 1 | 0.960 | 0.960 |
|  |  |  | CG | 118 (37.0) | 103 (36.4) | 1.01 (0.72-1.42) |  | 1.02 (0.72-1.43) |  |  |
|  |  |  | GG | 17 (5.3) | 17 (6.0) | 0.89 (0.44-1.79) |  | 0.92 (0.45-1.86) |  |  |
|  |  | Dominant | CC | 184 (57.7) | 163 (57.6) | 1 | 0.980 | 1 | 0.980 | 0.980 |
|  |  |  | CG/GG | 135 (42.3) | 120 (42.4) | 1.00 (0.72-1.38) |  | 1.00 (0.72-1.39) |  |  |
|  |  | Recessive | CC/CG | 302 (94.7) | 266 (94.0) | 1 | 0.720 | 1 | 0.790 | 0.865 |
|  |  |  | GG | 17 (5.3) | 17 (6.0) | 0.88 (0.44-1.76) |  | 0.91 (0.45-1.83) |  |  |
| rs10883512 | *HIF1AN* | Allele | A | 597 (93.3) | 518 (91.5) | - | 0.248 | - | - | - |
|  |  |  | G | 43 (6.7) | 48 (8.5) | - |  | - |  |  |
|  |  | Codominant | AA | 278 (86.9) | 238 (84.1) | 1 | 0.390 | 1 | 0.390 | 0.690 |
|  |  |  | AG | 41 (12.8) | 42 (14.8) | 0.84 (0.53-1.33) |  | 0.83 (0.52-1.32) |  |  |
|  |  |  | GG | 1 (0.3) | 3 (1.1) | 0.29 (0.03-2.76) |  | 0.29 (0.03-2.85) |  |  |
|  |  | Dominant | AA | 278 (86.9) | 238 (84.1) | 1 | 0.330 | 1 | 0.320 | 0.736 |
|  |  |  | AG/GG | 42 (13.1) | 45 (15.9) | 0.80 (0.51-1.26) |  | 0.79 (0.50-1.25) |  |  |
|  |  | Recessive | AA/AG | 319 (99.7) | 280 (98.9) | 1 | 0.250 | 1 | 0.260 | 0.544 |
|  |  |  | GG | 1 (0.3) | 3 (1.1) | 0.29 (0.03-2.83) |  | 0.30 (0.03-2.92) |  |  |
| rs4253623 | *PPARA* | Allele | A | 541 (85.6) | 491 (87.4) | - | 0.374 | - | - | - |
|  |  |  | G | 91 (14.4) | 71 (12.6) | - |  | - |  |  |
|  |  | Codominant | AA | 233 (73.8) | 212 (75.4) | 1 | 0.200 | 1 | 0.150 | 0.431 |
|  |  |  | AG | 75 (23.7) | 67 (23.8) | 1.02 (0.70-1.49) |  | 1.00 (0.68-1.47) |  |  |
|  |  |  | GG | 8 (2.5) | 2 (0.7) | 3.64 (0.76-17.32) |  | 4.05 (0.84-19.43) |  |  |
|  |  | Dominant | AA | 233 (73.8) | 212 (75.4) | 1 | 0.630 | 1 | 0.650 | 0.997 |
|  |  |  | AG/GG | 83 (26.2) | 69 (24.6) | 1.09 (0.76-1.58) |  | 1.09 (0.75-1.58) |  |  |
|  |  | Recessive | AA/AG | 308 (97.5) | 279 (99.3) | 1 | 0.072 | 1 | 0.051 | 0.235 |
|  |  |  | GG | 8 (2.5) | 2 (0.7) | 3.62 (0.76-17.19) |  | 4.05 (0.84-19.38) |  |  |
| rs135538 | *PPARA* | Allele | G | 350 (55.0) | 318 (56.2) | - | 0.688 | - | - | - |
|  |  |  | C | 286 (45.0) | 248 (43.8) | - |  | - |  |  |
|  |  | Codominant | GG | 102 (32.1) | 92 (32.5) | 1 | 0.850 | 1 | 0.800 | 0.920 |
|  |  |  | CG | 146 (45.9) | 134 (47.4) | 0.98 (0.68-1.42) |  | 1.00 (0.69-1.45) |  |  |
|  |  |  | CC | 70 (22.0) | 57 (20.1) | 1.11 (0.71-1.74) |  | 1.14 (0.73-1.80) |  |  |
|  |  | Dominant | GG | 102 (32.1) | 92 (32.5) | 1 | 0.910 | 1 | 0.810 | 0.932 |
|  |  |  | CG/CC | 216 (67.9) | 191 (67.5) | 1.02 (0.72-1.44) |  | 1.04 (0.74-1.47) |  |  |
|  |  | Recessive | GG/CG | 248 (78.0) | 226 (79.9) | 1 | 0.570 | 1 | 0.510 | 0.733 |
|  |  |  | CC | 70 (22.0) | 57 (20.1) | 1.12 (0.76-1.66) |  | 1.14 (0.77-1.70) |  |  |
| rs4253681 | *PPARA* | Allele | T | 504 (79.0) | 464 (81.7) | - | 0.241 | - | - | - |
|  |  |  | C | 134 (21.0) | 104 (18.3) | - |  | - |  |  |
|  |  | Codominant | TT | 197 (61.7) | 189 (66.5) | 1 | 0.470 | 1 | 0.440 | 0.675 |
|  |  |  | CT | 110 (34.5) | 86 (30.3) | 1.23 (0.87-1.73) |  | 1.24 (0.87-1.75) |  |  |
|  |  |  | CC | 12 (3.8) | 9 (3.2) | 1.28 (0.53-3.11) |  | 1.31 (0.54-3.21) |  |  |
|  |  | Dominant | TT | 197 (61.7) | 189 (66.5) | 1 | 0.220 | 1 | 0.200 | 0.767 |
|  |  |  | CT/CC | 122 (38.3) | 95 (33.5) | 1.23 (0.88-1.72) |  | 1.24 (0.89-1.74) |  |  |
|  |  | Recessive | TT/CT | 307 (96.2) | 275 (96.8) | 1 | 0.690 | 1 | 0.660 | 0.799 |
|  |  |  | CC | 12 (3.8) | 9 (3.2) | 1.19 (0.50-2.88) |  | 1.22 (0.50-2.97) |  |  |
| rs4253747 | *PPARA* | Allele | T | 496 (77.5) | 457 (80.7) | - | 0.168 | - | - | - |
|  |  |  | A | 144 (22.5) | 109 (19.3) | - |  | - |  |  |
|  |  | Codominant | TT | 194 (60.6) | 183 (64.7) | 1 | 0.280 | 1 | 0.270 | 0.565 |
|  |  |  | AT | 108 (33.8) | 91 (32.1) | 1.12 (0.79-1.58) |  | 1.15 (0.81-1.62) |  |  |
|  |  |  | AA | 18 (5.6) | 9 (3.2) | 1.89 (0.83-4.31) |  | 1.87 (0.81-4.27) |  |  |
|  |  | Dominant | TT | 194 (60.6) | 183 (64.7) | 1 | 0.310 | 1 | 0.260 | 0.748 |
|  |  |  | AT/AA | 126 (39.4) | 100 (35.3) | 1.19 (0.85-1.66) |  | 1.21 (0.87-1.69) |  |  |
|  |  | Recessive | TT/AT | 302 (94.4) | 274 (96.8) | 1 | 0.140 | 1 | 0.160 | 0.460 |
|  |  |  | AA | 18 (5.6) | 9 (3.2) | 1.81 (0.80-4.11) |  | 1.78 (0.78-4.04) |  |  |

^a^ adjusted for age, BMI and smoking status. ^*^*P*<0.05 indicted significant difference. “-” indicated “not available” for regression analysis or multiple hypothesis testing correction. *Q*-value was calculated using Benjamini and Hochberg method in multiple hypothesis testing including 23 SNPs for AMS association analysis. SNP. Single nucleotide polymorphism; AMS. Acute mountain sickness; BMI. Body mass index; *OR*. Odds ratio; CI. Confidence interval.
