# Supplementary material for: EPAS1 and VEGFA gene variants are related to the symptoms of acute mountain sickness in Chinese Han population: a cross-sectional study
Source: Mil Med Res. 2020 Jul 27;7:35. doi: 10.1186/s40779-020-00264-6 (PMC7385974; doi:10.1186/s40779-020-00264-6)
Supplement: Supplementary file 5 — Additional file 5: Table S4 .Associations between SNPs and AMS related-headache. [file 40779_2020_264_MOESM5_ESM.docx]

**Table S4**  Associations between SNPs and AMS-related headache

| SNP ID | Gene | Model | Allele/Genotype | HD group (*n =* 439) | Non-HD group (*n* = 165) | *OR* (95% CI) | *P*-value | *OR* (95% CI)^a^ | *P*-value^a^ | *Q*-value |
| --- | --- | --- | --- | --- | --- | --- | --- | --- | --- | --- |
| rs2153364 | *EGLN1* | Allele | A | 410 (51.4) | 163 (54.3) | - | 0.382 | - | - | - |
|  |  |  | G | 388 (48.6) | 137 (45.7) | - |  | - |  |  |
|  |  | Genotype | AA | 106 (26.6) | 47 (31.3) | 1 | 0.540 | 1 | 0.510 | 0.510 |
|  |  |  | AG | 198 (49.6) | 69 (46.0) | 1.27 (0.82-1.97) |  | 1.29 (0.83-2.01) |  |  |
|  |  |  | GG | 95 (23.8) | 34 (22.7) | 1.24 (0.74-2.09) |  | 1.24 (0.73-2.10) |  |  |
|  |  | Dominant | AA | 106 (26.6) | 47 (31.3) | 1 | 0.270 | 1 | 0.250 | 0.250 |
|  |  |  | AG/GG | 293 (73.4) | 103 (68.7) | 1.26 (0.84-1.90) |  | 1.28 (0.84-1.93) |  |  |
|  |  | Recessive | AA/AG | 304 (76.2) | 116 (77.3) | 1 | 0.780 | 1 | 0.810 | 0.810 |
|  |  |  | GG | 95 (23.8) | 34 (22.7) | 1.07 (0.68-1.67) |  | 1.06 (0.67-1.66) |  |  |
| rs6756667 | *EPAS1* | Allele | G | 785 (89.4) | 281 (85.2) | - | 0.041^*^ | - | - | - |
|  |  |  | A | 93 (10.6) | 49 (14.8) | - |  | - |  |  |
|  |  | Genotype | GG | 348 (79.3) | 120 (72.7) | 1 | 0.054 | 1 | 0.061 | 0.122 |
|  |  |  | GA | 89 (20.2) | 41 (24.9) | 0.75 (0.49-1.14) |  | 0.74 (0.48-1.14) |  |  |
|  |  |  | AA | 2 (0.5) | 4 (2.4) | 0.17 (0.03-0.95) |  | 0.18 (0.03-1.02) |  |  |
|  |  | Dominant | GG | 348 (79.3) | 120 (72.7) | 1 | 0.091 | 1 | 0.088 | 0.117 |
|  |  |  | AG/AA | 91 (20.7) | 45 (27.3) | 0.70 (0.46-1.05) |  | 0.69 (0.46-1.05) |  |  |
|  |  | Recessive | GG/AG | 437 (99.5) | 161 (97.6) | 1 | 0.044 | 1 | 0.053 | 0.212 |
|  |  |  | AA | 2 (0.5) | 4 (2.4) | 0.18 (0.03-1.02) |  | 0.20 (0.04-1.09) |  |  |
| rs3025039 | *VEGFA* | Allele | C | 722 (82.6) | 298 (90.3) | - | 0.001^*^ | - | - | - |
|  |  |  | T | 152 (17.4) | 32 (9.7) | - |  | - |  |  |
|  |  | Genotype | CC | 295 (67.5) | 134 (81.2) | 1 | 0.002* | 1 | 0.002^*^ | 0.008* |
|  |  |  | CT | 132 (30.2) | 30 (18.2) | 2.00 (1.28-3.12) |  | 1.99 (1.27-3.12) |  |  |
|  |  |  | TT | 10 (2.3) | 1 (0.6) | 4.54 (0.58-35.83) |  | 4.61 (0.58-36.60) |  |  |
|  |  | Dominant | CC | 295 (67.5) | 134 (81.2) | 1 | <0.001* | 1 | <0.001^*^ | <0.001* |
|  |  |  | CT/TT | 142 (32.5) | 31 (18.8) | 2.08 (1.34-3.23) |  | 2.08 (1.34-3.23) |  |  |
|  |  | Recessive | CC/CT | 427 (97.7) | 164 (99.4) | 1 | 0.130 | 1 | 0.120 | 0.240 |
|  |  |  | TT | 10 (2.3) | 1 (0.6) | 3.84 (0.49-30.23) |  | 3.90 (0.49-30.90) |  |  |
| rs7292407 | *PPARA* | Allele | C | 708 (85.9) | 261 (82.1) | - | 0.104 | - | - | - |
|  |  |  | A | 116 (14.1) | 57 (17.9) | - |  | - |  |  |
|  |  | Genotype | CC | 310 (75.2) | 107 (67.3) | 1 | 0.130 | 1 | 0.150 | 0.200 |
|  |  |  | AC | 88 (21.4) | 47 (29.6) | 0.65 (0.43-0.98) |  | 0.66 (0.43-1.00) |  |  |
|  |  |  | AA | 14 (3.4) | 5 (3.1) | 0.97 (0.34-2.75) |  | 1.05 (0.37-3.02) |  |  |
|  |  | Dominant | CC | 310 (75.2) | 107 (67.3) | 1 | 0.058 | 1 | 0.080 | 0.160 |
|  |  |  | AC/AA | 102 (24.8) | 52 (32.7) | 0.68 (0.45-1.01) |  | 0.70 (0.46-1.04) |  |  |
|  |  | Recessive | CC/AC | 398 (96.6) | 154 (96.9) | 1 | 0.880 | 1 | 0.750 | 0.810 |
|  |  |  | AA | 14 (3.4) | 5 (3.1) | 1.08 (0.38-3.06) |  | 1.18 (0.41-3.36) |  |  |

^a^ adjusted for age, BMI and smoking status. ^*^*P*<0.05 indicated significant difference. “-” indicated “not available” for regression analysis or multiple hypothesis testing correction. *Q*-value was calculated using Benjamini and Hochberg method in multiple hypothesis testing including above 4 SNPs. SNP. Single nucleotide polymorphism; AMS. Acute mountain sickness; HD. Headache; BMI. Body mass index; *OR*. Odds ratio; CI. Confidence interval.
