# Supplementary material for: EPAS1 and VEGFA gene variants are related to the symptoms of acute mountain sickness in Chinese Han population: a cross-sectional study
Source: Mil Med Res. 2020 Jul 27;7:35. doi: 10.1186/s40779-020-00264-6 (PMC7385974; doi:10.1186/s40779-020-00264-6)
Supplement: Supplementary file 6 — Additional file 6: Table S5. Associations between SNPs and AMS related-dizziness/light-headedness. [file 40779_2020_264_MOESM6_ESM.docx]

**Table S5** Associations between SNPs and AMS-related dizziness/light-headedness

| SNP ID | Gene | Model | Allele/Genotype | DL group (*n*=433) | Non-DL group (*n*=171) | *OR* (95% CI) | *P*-value | *OR* (95% CI)^a^ | *P*-value^a^ | *Q*-value |
| --- | --- | --- | --- | --- | --- | --- | --- | --- | --- | --- |
| rs2153364 | *EGLN1* | Allele | A | 399 (51.2) | 174 (54.7) | - | 0.284 | - | - | - |
|  |  |  | G | 381 (48.8) | 144 (45.3) | - |  | - |  |  |
|  |  | Genotype | AA | 101 (25.9) | 52 (32.7) | 1 | 0.240 | 1 | 0.220 | 0.220 |
|  |  |  | AG | 197 (50.5) | 70 (44.0) | 1.45 (0.94-2.23) |  | 1.48 (0.96-2.28) |  |  |
|  |  |  | GG | 92 (23.6) | 37 (23.3) | 1.28 (0.77-2.13) |  | 1.28 (0.77-2.14) |  |  |
|  |  | Dominant | AA | 101 (25.9) | 52 (32.7) | 1 | 0.110 | 1 | 0.098 | 0.098 |
|  |  |  | AG/GG | 289 (74.1) | 107 (67.3) | 1.39 (0.93-2.08) |  | 1.41 (0.94-2.11) |  |  |
|  |  | Recessive | AA/AG | 298 (76.4) | 122 (76.7) | 1 | 0.940 | 1 | 0.970 | 0.970 |
|  |  |  | GG | 92 (23.6) | 37 (23.3) | 1.02 (0.66-1.57) |  | 1.01 (0.65-1.56) |  |  |
| rs6756667 | *EPAS1* | Allele | G | 776 (89.6) | 290 (84.8) | - | 0.019^*^ | - | - | - |
|  |  |  | A | 90 (10.4) | 52 (15.2) | - |  | - |  |  |
|  |  | Genotype | GG | 344 (79.5) | 124 (72.5) | 1 | 0.008* | 1 | 0.010 | 0.040 |
|  |  |  | GA | 88 (20.3) | 42 (24.6) | 0.76 (0.50-1.15) |  | 0.76 (0.50-1.16) |  |  |
|  |  |  | AA | 1 (0.2) | 5 (2.9) | 0.07 (0.01-0.62) |  | 0.08 (0.01-0.66) |  |  |
|  |  | Dominant | GG | 344 (79.5) | 124 (72.5) | 1 | 0.070 | 1 | 0.077 | 0.154 |
|  |  |  | AG/AA | 89 (20.6) | 47 (27.5) | 0.68 (0.45-1.03) |  | 0.69 (0.46-1.04) |  |  |
|  |  | Recessive | GG/AG | 432 (99.8) | 166 (97.1) | 1 | 0.005* | 1 | 0.006* | 0.024* |
|  |  |  | AA | 1 (0.2) | 5 (2.9) | 0.08 (0.01-0.66) |  | 0.08 (0.01-0.70) |  |  |
| rs3025039 | *VEGFA* | Allele | C | 720 (83.5) | 300 (87.7) | - | 0.068 | - | - | - |
|  |  |  | T | 142 (16.5) | 42 (12.3) | - |  | - |  |  |
|  |  | Genotype | CC | 299 (69.4) | 130 (76.0) | 1 | 0.110 | 1 | 0.094 | 0.188 |
|  |  |  | CT | 122 (28.3) | 40 (23.4) | 1.33 (0.88-2.00) |  | 1.36 (0.90-2.06) |  |  |
|  |  |  | TT | 10 (2.3) | 1 (0.6) | 4.35(0.55-34.30) |  | 4.44 (0.56-35.14) |  |  |
|  |  | Dominant | CC | 299 (69.4) | 130 (76.0) | 1 | 0.100 | 1 | 0.080 | 0.107 |
|  |  |  | CT/TT | 132 (30.6) | 41 (24.0) | 1.40 (0.93-2.10) |  | 1.43 (0.95-2.16) |  |  |
|  |  | Recessive | CC/CT | 421 (97.7) | 170 (99.4) | 1 | 0.110 | 1 | 0.110 | 0.220 |
|  |  |  | TT | 10 (2.3) | 1 (0.6) | 4.04 (0.51-31.78) |  | 4.10 (0.52-32.31) |  |  |
| rs7292407 | *PPARA* | Allele | C | 698 (86.2) | 271 (81.6) | - | 0.052 | - | - | - |
|  |  |  | A | 112 (13.8) | 61 (18.4) | - |  | - |  |  |
|  |  | Genotype | CC | 304 (75.1) | 113 (68.1) | 1 | 0.180 | 1 | 0.140 | 0.187 |
|  |  |  | AC | 90 (22.2) | 45 (27.1) | 0.74 (0.49-1.13) |  | 0.72 (0.47-1.09) |  |  |
|  |  |  | AA | 11 (2.7) | 8 (4.8) | 0.51 (0.20-1.30) |  | 0.50 (0.19-1.28) |  |  |
|  |  | Dominant | CC | 304 (75.1) | 113 (68.1) | 1 | 0.091 | 1 | 0.066 | 0.264 |
|  |  |  | AC/AA | 101 (24.9) | 53 (31.9) | 0.71 (0.48-1.05) |  | 0.68 (0.46-1.02) |  |  |
|  |  | Recessive | CC/AC | 394 (97.3) | 158 (95.2) | 1 | 0.220 | 1 | 0.210 | 0.280 |
|  |  |  | AA | 11 (2.7) | 8 (4.8) | 0.55 (0.22-1.40) |  | 0.54 (0.21-1.38) |  |  |

^a^ adjusted for age, BMI and smoking status. * *P*<0.05 indicated significant difference. “-” indicated “not available” for regression analysis or multiple hypothesis testing correction. *Q*-value was calculated using Benjamini and Hochberg method in multiple hypothesis testing including above 4 SNPs. SNP. Single nucleotide polymorphism; AMS. Acute mountain sickness; DL. Dizziness and light-headedness; BMI. Body mass index; *OR*. Odds ratio; CI. Confidence interval.
