# Supplementary material for: EPAS1 and VEGFA gene variants are related to the symptoms of acute mountain sickness in Chinese Han population: a cross-sectional study
Source: Mil Med Res. 2020 Jul 27;7:35. doi: 10.1186/s40779-020-00264-6 (PMC7385974; doi:10.1186/s40779-020-00264-6)
Supplement: Supplementary file 7 — Additional file 7: Table S6. Associations between SNPs and AMS related-gastrointestinal symptoms. [file 40779_2020_264_MOESM7_ESM.docx]

**Table S6** Associations between SNPs and AMS-related gastrointestinal symptoms

| SNP ID | Gene | Model | Allele/Genotype | GI group (*n* = 134) | Non-GI group (*n =* 470) | *OR* (95% CI) | *P*-value | *OR* (95% CI)^a^ | *P*-value^a^ | *Q*-value |
| --- | --- | --- | --- | --- | --- | --- | --- | --- | --- | --- |
| rs2153364 | *EGLN1* | Allele | A | 124 (51.2) | 449 (52.5) | - | 0.739 | - | - | - |
|  |  |  | G | 118 (48.8) | 407 (47.5) | - |  | - |  |  |
|  |  | Genotype | AA | 29 (24.0) | 124 (29.0) | 1 | 0.330 | 1 | 0.310 | 0.413 |
|  |  |  | AG | 66 (54.5) | 201 (47.0) | 1.40 (0.86-2.29) |  | 1.41 (0.86-2.31) |  |  |
|  |  |  | GG | 26 (21.5) | 103 (24.1) | 1.08 (0.60-1.95) |  | 1.07 (0.59-1.93) |  |  |
|  |  | Dominant | AA | 29 (24.0) | 124 (29.0) | 1 | 0.270 | 1 | 0.270 | 0.540 |
|  |  |  | AG/GG | 92 (76.0) | 304 (71.0) | 1.29 (0.81-2.06) |  | 1.29 (0.81-2.07) |  |  |
|  |  | Recessive | AA/AG | 95 (78.5) | 325 (75.9) | 1 | 0.550 | 1 | 0.510 | 0.680 |
|  |  |  | GG | 26 (21.5) | 103 (24.1) | 0.86 (0.53-1.41) |  | 0.85 (0.52-1.39) |  |  |
| rs6756667 | *EPAS1* | Allele | G | 248 (92.5) | 818 (87.0) | - | 0.013^*^ | - | - | - |
|  |  |  | A | 20 (7.5) | 122 (13.0) | - |  | - |  |  |
|  |  | Genotype | GG | 114 (85.1) | 354 (75.3) | 1 | 0.018* | 1 | 0.018^*^ | 0.072 |
|  |  |  | GA | 20 (14.9) | 110 (23.4) | 0.56 (0.34-0.95) |  | 0.56 (0.33-0.94) |  |  |
|  |  |  | AA | 0 (0.0) | 6 (1.3) | 0.00 (0.00-NA) |  | 0.00 (0.00-NA) |  |  |
|  |  | Dominant | GG | 114 (85.1) | 354 (75.3) | 1 | 0.014* | 1 | 0.013^*^ | 0.052 |
|  |  |  | AG/AA | 20 (14.9) | 116 (24.7) | 0.54 (0.32-0.90) |  | 0.53 (0.32-0.89) |  |  |
|  |  | Recessive | GG/AG | 134 (100.0) | 464 (98.7) | 1 | 0.082 | 1 | 0.092 | 0.184 |
|  |  |  | AA | 0 (0.0) | 6 (1.3) | 0.00 (0.00-NA) |  | 0.00 (0.00-NA) |  |  |
| rs3025039 | *VEGFA* | Allele | C | 225 (84.6) | 795 (84.8) | - | 0.946 | - | - | - |
|  |  |  | T | 41 (15.4) | 143 (15.2) | - |  | - |  |  |
|  |  | Genotype | CC | 95 (71.4) | 334 (71.2) | 1 | 0.910 | 1 | 0.910 | 0.910 |
|  |  |  | CT | 35 (26.3) | 127 (27.1) | 0.97 (0.63-1.50) |  | 0.97 (0.62-1.50) |  |  |
|  |  |  | TT | 3 (2.3) | 8 (1.7) | 1.32 (0.34-5.07) |  | 1.33 (0.34-5.13) |  |  |
|  |  | Dominant | CC | 95 (71.4) | 334 (71.2) | 1 | 0.960 | 1 | 0.950 | 0.950 |
|  |  |  | CT/TT | 38 (28.6) | 135 (28.8) | 0.99 (0.65-1.52) |  | 0.99 (0.64-1.51) |  |  |
|  |  | Recessive | CC/CT | 130 (97.7) | 461 (98.3) | 1 | 0.680 | 1 | 0.680 | 0.680 |
|  |  |  | TT | 3 (2.3) | 8 (1.7) | 1.33 (0.35-5.08) |  | 1.34 (0.35-5.16) |  |  |
| rs7292407 | *PPARA* | Allele | C | 223 (87.8) | 746 (84.0) | - | 0.138 | - | - | - |
|  |  |  | A | 31 (12.2) | 142 (16.0) | - |  | - |  |  |
|  |  | Genotype | CC | 97 (76.4) | 320 (72.1) | 1 | 0.100 | 1 | 0.120 | 0.240 |
|  |  |  | AC | 29 (22.8) | 106 (23.9) | 0.90 (0.56-1.44) |  | 0.92 (0.57-1.47) |  |  |
|  |  |  | AA | 1 (0.8) | 18 (4.0) | 0.18 (0.02-1.39) |  | 0.19 (0.03-1.46) |  |  |
|  |  | Dominant | CC | 97 (76.4) | 320 (72.1) | 1 | 0.330 | 1 | 0.370 | 0.493 |
|  |  |  | AC/AA | 30 (23.6) | 124 (27.9) | 0.80 (0.50-1.26) |  | 0.81 (0.51-1.29) |  |  |
|  |  | Recessive | CC/AC | 126 (99.2) | 426 (96.0) | 1 | 0.037* | 1 | 0.045^*^ | 0.180 |
|  |  |  | AA | 1 (0.8) | 18 (4.0) | 0.19 (0.02-1.42) |  | 0.20 (0.03-1.49) |  |  |

^a^ adjusted for age, BMI and smoking status. ^*^*P*<0.05 indicated significant difference. “-” indicated “not available” for regression analysis or multiple hypothesis testing correction. *Q*-value was calculated using Benjamini and Hochberg method in multiple hypothesis testing including above 4 SNPs. SNP. Single nucleotide polymorphism; AMS. Acute mountain sickness; GI. Gastrointestinal symptoms; *OR*. Odds ratio; CI. Confidence interval.
