# Supplementary material for: EPAS1 and VEGFA gene variants are related to the symptoms of acute mountain sickness in Chinese Han population: a cross-sectional study
Source: Mil Med Res. 2020 Jul 27;7:35. doi: 10.1186/s40779-020-00264-6 (PMC7385974; doi:10.1186/s40779-020-00264-6)
Supplement: Supplementary file 8 — Additional file 8: Table S7. Associations between SNPs and AMS related-fatigue. [file 40779_2020_264_MOESM8_ESM.docx]

**Table S7** Associations between SNPs and AMS-related fatigue

| SNP ID | Gene | Model | Allele/Genotype | FA group (*n =* 412) | Non-FA group (*n* = 192) | *OR* (95% CI) | *P*-value | *OR* (95% CI)^a^ | *P*-value^a^ | *Q*-value |
| --- | --- | --- | --- | --- | --- | --- | --- | --- | --- | --- |
| rs2153364 | *EGLN1* | Allele | A | 381 (51.2) | 192 (54.2) | - | 0.348 | - | - | - |
|  |  |  | G | 363 (48.8) | 162 (45.8) | - |  | - |  |  |
|  |  | Genotype | AA | 98 (26.3) | 55 (31.1) | 1 | 0.520 | 1 | 0.470 | 0.470 |
|  |  |  | AG | 185 (49.7) | 82 (46.3) | 1.27 (0.83-1.93) |  | 1.28 (0.84-1.96) |  |  |
|  |  |  | GG | 89 (23.9) | 40 (22.6) | 1.25 (0.76-2.06) |  | 1.29 (0.78-2.13) |  |  |
|  |  | Dominant | AA | 98 (26.3) | 55 (31.1) | 1 | 0.250 | 1 | 0.220 | 0.220 |
|  |  |  | AG/GG | 274 (73.7) | 122 (68.9) | 1.26 (0.85-1.87) |  | 1.28 (0.86-1.91) |  |  |
|  |  | Recessive | AA/AG | 283 (76.1) | 137 (77.4) | 1 | 0.730 | 1 | 0.650 | 0.650 |
|  |  |  | GG | 89 (23.9) | 40 (22.6) | 1.08 (0.70-1.65) |  | 1.10 (0.72-1.69) |  |  |
| rs6756667 | *EPAS1* | Allele | G | 738 (89.6) | 328 (85.4) | - | 0.037^*^ | - | - | - |
|  |  |  | A | 86 (10.4) | 56 (14.6) | - |  | - |  |  |
|  |  | Genotype | GG | 328 (79.6) | 140 (72.9) | 1 | 0.068 | 1 | 0.067 | 0.089 |
|  |  |  | GA | 82 (19.9) | 48 (25.0) | 0.73 (0.49-1.10) |  | 0.72 (0.48-1.09) |  |  |
|  |  |  | AA | 2 (0.5) | 4 (2.1) | 0.21 (0.04-1.18) |  | 0.21 (0.04-1.18) |  |  |
|  |  | Dominant | GG | 328 (79.6) | 140 (72.9) | 1 | 0.070 | 1 | 0.066 | 0.264 |
|  |  |  | AG/AA | 84 (20.4) | 52 (27.1) | 0.69 (0.46-1.03) |  | 0.68 (0.46-1.02) |  |  |
|  |  | Recessive | GG/AG | 410 (99.5) | 188 (97.9) | 1 | 0.079 | 1 | 0.080 | 0.107 |
|  |  |  | AA | 2 (0.5) | 4 (2.1) | 0.23 (0.04-1.26) |  | 0.23 (0.04-1.27) |  |  |
| rs3025039 | *VEGFA* | Allele | C | 682 (83.2) | 338 (88.0) | - | 0.029^*^ | - | - | - |
|  |  |  | T | 138 (16.8) | 46 (12.0) | - |  | - |  |  |
|  |  | Genotype | CC | 283 (69.0) | 146 (76.0) | 1 | 0.006* | 1 | 0.007^*^ | 0.014^*^ |
|  |  |  | CT | 116 (28.3) | 46 (24.0) | 1.30 (0.88-1.93) |  | 1.28 (0.86-1.91) |  |  |
|  |  |  | TT | 11 (2.7) | 0 (0.0) | NA (0.00-NA) |  | NA (0.00-NA) |  |  |
|  |  | Dominant | CC | 283 (69.0) | 146 (76.0) | 1 | 0.073 | 1 | 0.089 | 0.178 |
|  |  |  | CT/TT | 127 (31.0) | 46 (24.0) | 1.42 (0.96-2.11) |  | 1.40 (0.95-2.08) |  |  |
|  |  | Recessive | CC/CT | 399 (97.3) | 192 (100.0) | 1 | 0.004* | 1 | 0.004^*^ | 0.008^*^ |
|  |  |  | TT | 11 (2.7) | 0 (0.0) | NA (0.00-NA) |  | NA (0.00-NA) |  |  |
| rs7292407 | *PPARA* | Allele | C | 672 (86.8) | 297 (80.7) | - | 0.007^*^ | - | - | - |
|  |  |  | A | 102 (13.2) | 71 (19.3) | - |  | - |  |  |
|  |  | Genotype | CC | 291 (75.2) | 126 (68.5) | 1 | 0.004* | 1 | 0.006^*^ | 0.024^*^ |
|  |  |  | AC | 90 (23.3) | 45 (24.5) | 0.87 (0.57-1.31) |  | 0.88 (0.58-1.33) |  |  |
|  |  |  | AA | 6 (1.6) | 13 (7.1) | 0.20 (0.07-0.54) |  | 0.21 (0.08-0.58) |  |  |
|  |  | Dominant | CC | 291 (75.2) | 126 (68.5) | 1 | 0.094 | 1 | 0.120 | 0.160 |
|  |  |  | AC/AA | 96 (24.8) | 58 (31.5) | 0.72 (0.49-1.06) |  | 0.73 (0.49-1.08) |  |  |
|  |  | Recessive | CC/AC | 381 (98.5) | 171 (92.9) | 1 | 0.001* | 1 | 0.002* | 0.008* |
|  |  |  | AA | 6 (1.6) | 13 (7.1) | 0.21 (0.08-0.55) |  | 0.22 (0.08-0.59) |  |  |

^a^ adjusted for age, BMI and smoking status. * *P*<0.05 indicated significant difference. “-” indicated “not available” for regression analysis or multiple hypothesis testing correction. *Q*-value was calculated using Benjamini and Hochberg method in multiple hypothesis testing including above 4 SNPs. SNP. Single nucleotide polymorphism; AMS. Acute mountain sickness; FA. Fatigue and/or weakness; *OR*. Odds ratio; CI. Confidence interval.
